# Conditional Disease Development extracted from Longitudinal Health Care Cohort Data using Layered Network Construction

**DOI:** 10.1038/srep26170

**Published:** 2016-05-23

**Authors:** Venkateshan Kannan, Fredrik Swartz, Narsis A. Kiani, Gilad Silberberg, Giorgos Tsipras, David Gomez-Cabrero, Kristina Alexanderson, Jesper Tegnèr

**Affiliations:** 1 Computational Medicine Unit, Department of Medicine, Solna, Karolinska Institutet, SE-17176, Stockholm, Sweden; 2 Center for Molecular Medicine, L8:05, SE-17176, Stockholm, Karolinska Institutet, Sweden; 3 Division of Insurance Medicine, Department of Clinical Neuroscience, Karolinska Institutet, SE-17177 Stockholm, Sweden; 4 Unit of Clinical Epidemiology, Department of Medicine, Karolinska University Hospital L8, SE-17176, Stockholm, Sweden; 5 Science for Life Laboratory, Stockholm, Sweden

## Abstract

Health care data holds great promise to be used in clinical decision support systems. However, frequent near-synonymous diagnoses recorded separately, as well as the sheer magnitude and complexity of the disease data makes it challenging to extract non-trivial conclusions beyond confirmatory associations from such a web of interactions. Here we present a systematic methodology to derive statistically valid conditional development of diseases. To this end we utilize a cohort of 5,512,469 individuals followed over 13 years at inpatient care, including data on disability pension and cause of death. By introducing a causal information fraction measure and taking advantage of the composite structure in the ICD codes, we extract an effective directed lower dimensional network representation (100 nodes and 130 edges) of our cohort. Unpacking composite nodes into bipartite graphs retrieves, for example, that individuals with behavioral disorders are more likely to be followed by prescription drug poisoning episodes, whereas women with leiomyoma were more likely to subsequently experience endometriosis. The conditional disease development represent putative causal relations, indicating possible novel clinical relationships and pathophysiological associations that have not been explored yet.

The success of personalized medicine depends on the existence of clinically practical tools that can assess the individual’s risk of diseases and suggest an efficient therapy at the right time. Yet, despite the fact that this is an overarching ambition driving numerous current efforts in translational research it has proven very difficult to realize in practice. Using population wide data to analyze disease correlations and patterns of comorbidities has recently emerged as a promising path towards extracting relationships that could be used in clinical trials whose outcomes, depending on the results, can be encoded in clinical decision support systems in healthcare. Statistical methods such as those proposed in the current paper serve to facilitate and inform the selection of the most likely candidates to be brought to confirmation in a clinical trial. When augemnted with molecular information we may even discover new molecular mechanisms mediating comorbidity and correlation patterns between diseases^1^,^2^,^3^. Indeed, numerous recent studies have utilized a large-scale system based approach to address disease associations, symptoms, transitions^4^,^5^,^6^,^7^ and a corresponding molecular systems analysis^8^,^9^,^10^. The relationship between networks arising from different data types in turn provides complimentary insights into how disease interactions are mediated at different levels^11^. A common denominator of this body of exciting work is the aim to advance beyond an epidemiological or molecular analysis of a single disease or a small set of diseases in isolation, in order to arrive at systems understanding with the idea of enabling personalized medicine. Yet, this approach comes with the price of dealing with data of daunting complexity and size, which in turn constitutes a challenging barrier to overcome in order to derive rigorous and non-trivial conclusions. The analyses of such dense networks of nodes (in thousands) and edges (in hundreds of thousands) have on the one hand provided interesting observations and associations but yet has proven difficult to extract statistically valid specific predictive relationships.

In this context, we develop and present a systematic methodology to derive statistically sound conditional development between diseases over time. We identify putative causal relations which subsequently can be further interrogated either from a clinical perspective asking how the clinical context can induce the derived relation or the prediction could be assessed from a molecular vantage point thereby dissecting the biological underpinnings driving the association. To this end we base our methodology and analysis on the data from a cohort of all the 5.5 million individuals aged between 16 and 64 as of Dec 31, 1994 in Sweden. This cohort was followed with data from high quality, nationwide registries for 13 consecutive years (1997–2009). As we recognize that a graph based analysis of the raw cohort data suffers from the limitations mentioned above we have developed a three-step procedure to arrive at the underlying core of the data-set. We refer to this as an effective low-dimensional representation of the full data-set, in the sense of capturing the most interesting and non-trivial associations. To elaborate on this point, we first observe, as has been reported previously, that our cohort captures several known and interesting statistics on a population level such as age and gender distribution. Yet, to assess whether the cohort contains strong signals, which are statistically different from random, we derive test-statistics and appropriate null distributions that proves that this is indeed the case, thus justifying the utilization of this cohort as a vehicle for developing a systematic method.

Our three-step procedure runs as follows: first, the data-set is enriched with more specific information which takes directional nature into account by introducing a causal information fraction measure, thus representing putative causal relationships. Next, the inherent composite structure in the ICD codes is utilized, such that similar diseases are collapsed into composite diseases, thereby allowing a construction of a lower-dimensional network, effectively reducing the number of nodes from 1400+ to less than 200. This procedure mitigates the effect of overcrowding of the network nodes. Finally, we zoom in at an intermediate range of this network by introducing a lower and higher threshold on the edges. This is guided by the observation that for an edge to make a difference it should have significant non-zero value while not being too strong to render it biologically trivial. This procedure results in an effective, directed, lower dimensional network representation (99 nodes and 130 edges) of our cohort.

Using strategies such as unpacking interesting composite nodes into bipartite graphs, we retrieve known relationships thus validating our approach as well as identifying novel statistically predictive relationships. Overall, hubs refer to vertices with relatively large number edges, and in-degree refers to the number of incoming edges. Thus, in-degree hubs are those hubs where the ratio of incoming to outgoing edges is very large. Therefore, in this reduced network representation the in-degree-hubs are, as expected, identified as symptoms of a number of related conditions. More specifically, we recover, among other things, a well-known fact that various lymphomas are followed by diagnosis of anemia and purpura. Having validated our directed network, we turn to associations in our data-set that are less well-established, for example, that individuals with behavioral disorders are more likely to have prescription drug poisoning episodes than any other major disease. In another association, abnormal findings in blood plasma (serum enzyme levels, white blood cells, viscosity) are identified as statistically novel predictors for Type II diabetes. Finally we perform a targeted analysis of the relationships between autoimmune diseases and different types of tumors. Here we find that autoimmune diseases cluster more tightly compared to cancers, and are also more likely to form in-degree hubs. Intriguingly, some tumors are predictors for autoimmune disease such Crohn’s Disease, Type I diabetes, and psoriasis.

## Results

The result section is organized in three parts. First, we illustrate that the cohort data is sound by inspecting age, gender, and patterns of diagnosis. To ensure that the cohort data contains a statistically significant signal, we derive null distributions and test statistics. Secondly, we motivate and develop our three-step methodology, which takes direction and the layered structure of the data into account. Finally, we illustrate known and novel statistical predictive relationships between diseases.

We consider patterns of age and gender dependencies and find that the average number of diagnoses increases with age in the cohort overall except around the age of 30, when pregnancy-related complications spike in women (Supplement Section C and Supplement Fig. 2). For more gender-related effects, we note that the average number of diagnoses overall is about 1.25 times greater in women than in men. Further analysis (Supplement Section C) shows effects that are consistent with our standard understanding of the relationship of specific disease categories like autoimmune disorders with gender.

We use the statistics of the overall data to show that (a) there is a very strong and unambiguous positive association among diseases and (b) data is meaningful, coherent and consistent. In the process, we directly arrive at p-values for disease co-occurrence, and by using the combinatorial identities to show that patients with *k* previous diagnosis are far more likely to have a (*k* + 1)’th diagnosis than those individuals with fewer diseases.

### Aggregate Data Analysis

#### Comborbidity Distribution

We use relative risk (RR) to measure comorbidity, defined as


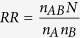


where *n*_*A*_ and *n*_*B*_ are the prevalences, i.e., the number of individuals diagnosed with diseases *A* and *B* respectively, and *n*_*AB*_ is the number that have both, and *N* is the total cohort population. Greater the value of RR, more is the association, and a value of unity represents no association. We want to find out how significant the values of RR in our data-set are.

**Figure 1:**
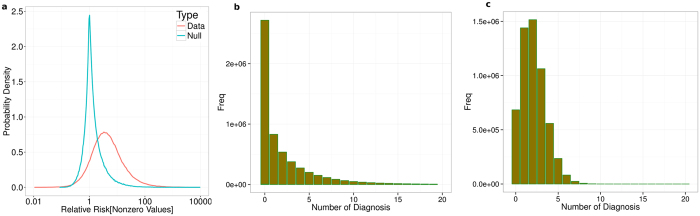
(**a**) Plot of the distribution of RR for the data-set and the null set obtained using the method described in Supplement A. (**b**,**c**) Comparison of the disease diagnoses frequency from the data (**b**) and the null distribution (**c**) given by Eq. (1) (see Methods section).

In Supplement A, we prove that, for a given pair of diseases with prevalences *n* and *m*, the co-occurrences, assuming independence of the diseases, follow Poisson distribution with parameter 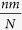, for sufficiently large prevalences. Sampling from the Poisson distribution, we generate a co-occurrence for every pair of diseases, and RR is calculated using this. The null distribution is the collection of values of RR from all such pairwise computations (~600,000). Figure 1a shows the two distributions where we find that the cohort curve is much wider, asymmetric with a heavy tail on the right, pointing unambiguously to strong positive associations within the data. Indeed the quantity 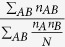 that compares sum of all pairwise co-occurrences with that expected by chance, is as great as 3.26 (*p* < 10^−27^).

Furthermore, Poisson distribution of co-occurrences directly gives a p-value for our data, unlike the approach in^5^, where the p-value is computed indirectly for the RR and Φ measure based on other approximations.

#### Disease Diagnosis Frequency

We turn towards disease diagnoses frequency and Fig. 1b,c show the cohort distribution and the null distribution (described in Methods) side by side. The considerable divergence between the two, and specifically the heavy tailed distribution of the cohort, shows that (a) disease accumulation follows a power-law and that (b) the likelihood of developing *k* + 1’th disease following *k* previous diagnoses is considerably greater than what would be expected if the disease occurrences were random. Other analysis considering only a limited number of simultaneous diseases showed similar trends^12^. We would like to add that, while this represents an important insight into disease accumulation, our disease dataset consists of only pairwise associations (which does not permit examining this property).

### Networks

**Figure 2:**
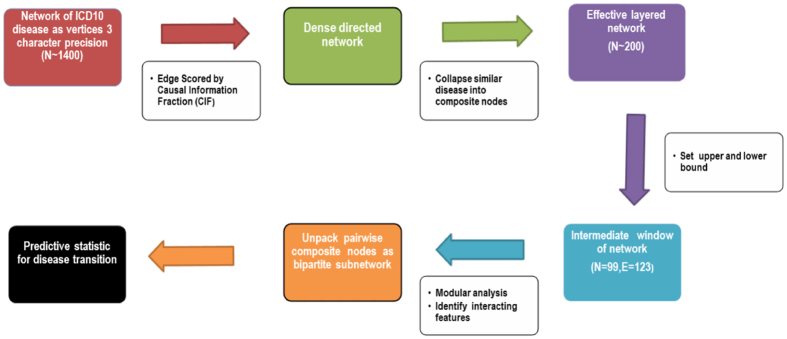
Schematic of the approach to our analysis pipeline of the cohort data. Starting from all ICD 10 classified three-character diseases (N ~ 1400+), we collapse similar diseases into composite node and construct a lower dimensional, effective layered network using a directed measure for the edges. We prune the network through lower and upper bounds on the measure, and examine the resulting intermediate window of network. Edges containing significant new information are further inspected by unpacking pairwise composite nodes into a bipartite directed network. These lead to putative causal associations at the three character level that is of most interest to us.

As noted and shown in previous works^4^,^5^,^6^, the networks generated on disease nodes are typically complex and dense, regardless of the specific choice of measures or other parameter tuning. As such for a set of *K* diseases, there are in all 
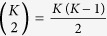
 possible associations, and with *K* ∼ 10^3^, this gives a massive ~10^6^ pairs. Although they potentially contain plenty of useful information, it requires development of specialized techniques to be able to identify the interesting links that have significant relevance biologically. We developed a systematic layered procedure that examines the interaction structure at two different scales to overcome the problem. The following steps give an outline of the procedure: (a) devise a directed pairwise measure that captures putative causal interactions (b) collapsing nodes of similar diseases into a composite node, resulting in a smaller and far more accessible network (c) setting an upper bound on the measure to eliminate the strongest of interactions and (d) investigate the interesting edges by unpacking them into a bipartite network. The schematic is given in Fig. 2.

In previous work^5^, the main choice of measures were relative risk and the *ϕ* correlation coefficient. In addition to the fact that these have known biases, the fundamental drawback about them is that they are symmetric measures that lead to undirected edges in the network. Adopting one of these measures leads to ignoring an important variable in our data-set: the order of occurrence for a given pair of diseases.

Thus our first step was formulating a new measure that determined how diseases progressed from one to the other. We define the Causal Information Fraction (CIF) between a pair of diseases *i* and *j* with prevalences *n*_*i*_ and *n*_*j*_ respectively:


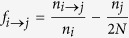


where *n*_*i*→*j*_ is the number of individuals that had disease *i* were later diagnosed with disease *j*. Although any inference using this or other measures based only on our data-set does not - and cannot - ascertain causation, this measure aims to emphasize possible causative (direct or indirect) effects between disease pairs. Specifically, as opposed to the family of measures associated to odds ratio, which is based on ratios of probability to develop a disease with or without some exposure condition, this places weight on the fraction that go on to develop that disease.

As in previous works, we confirm that the full network on 1400+ diseases of three-character ICD 10 classification^13^, leads to a highly dense topology (Supplement Figs 5 and 6 show networks of average degrees of 20 and 5) that is impossible to make sense of visually. In addition to clarity, this network it too opaque to identify either the truly relevant edges or characterizing the inherent structure of the network. While there are network-theoretic tools probing different characteristics of the network and identifying nodes and edges of specific significance, we note that these by themselves are not particularly interesting or informative (Supplement Section E describes limitations of standard network tools). We believe that is part of the explanation for why previous studies were more descriptive about the network but were relatively thin on actual novel biological inferences.

The second step in our methodology was guided by a need to provide a wider-scale view of the network. We wanted to treat diseases that are similar to each other as a single entity and construct edges upon those newly defined entities. We achieved this using the existing approximate classification implicit in the ICD 10 disease codes. In general, the subset of 3-character ICD 10 coded diseases of the form ‘Ω × [0–9]’, where Ω× is fixed (Ω is an alphabet and × is a decimal) and [0–9] indicates a single selection between 0 and 9, were collapsed to a single ‘Ω×’ composite node, and the corresponding statistics were calculated independently for every pair of such composite nodes(see Methods for details). Following this procedure leads to a collapse from 1400+ nodes to about 200 composite nodes.

With these composite nodes comprising the new vertices, we followed an identical procedure as before to construct a network by drawing edges between two vertices if the CIF measure is greater than a set threshold. Although this simplifies the network topology considerably, we discovered that the strongest of interactions are also those that are trivial and obvious (see Supplement Section F for examples). This is not very unusual because purely data-driven methods such as ours tend to highlight straightforward and unambiguous associations. Indeed the fact that we have several examples of obvious causal associations is a natural validation of our metric which aims to uncover putative causal structure.

**Figure 3:**
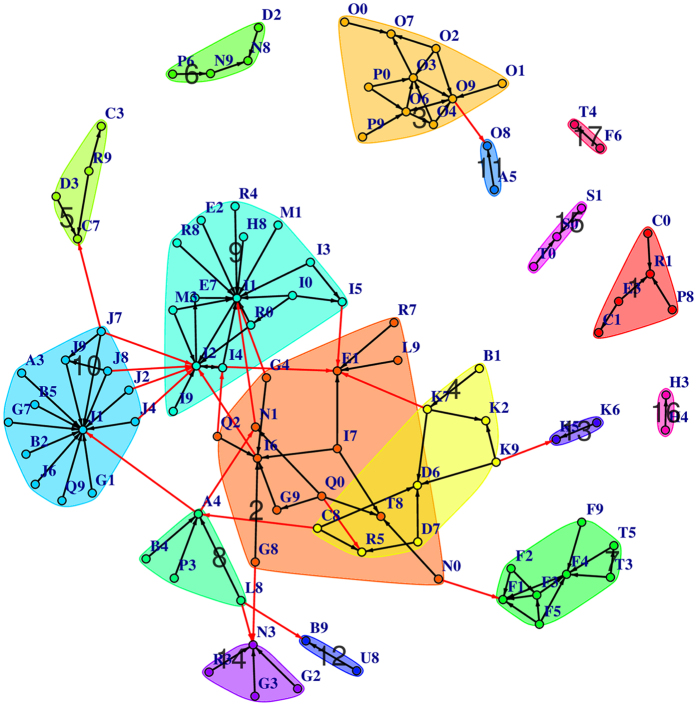
Directed network constructed on composite disease nodes based on the causal metric with both upper and lower bounds on the CIF measure (absolute values 0.21 and 0.15, respectively). The bounded regions marked by overlaid color correspond to the different clusters identified by community detection algorithm. The cluster number is marked against the cluster from 1 to 17. Number of vertices and edges is 99 and 130 respectively. The red edges link nodes in different clusters. ICD 10 Chapter Titles and their corresponding code ranges are given in Supplement Section M.

However, we are interested in associations that are not manifestly true. Hence, the third (and counter-intuitive) step was introducing an upper bound on the CIF measure leading to a focus on interactions of intermediate strengths, avoiding both those that are trivially true and those that are uninterestingly weak. We emphasize overall network structural clarity and identification of non-trivial association and this led us to the network shown in in Fig. 3 for detailed analysis.

The bounds for the network was set by in terms of the average degree of all composite nodes (isolated nodes have been removed from Fig. 3). With that index, the network in Fig. 3 was obtained with upper and lower bounds of average degree 0.8 and 2.1 respectively (see Supplement Section K for details on the stability of these choices).

Figure 3 also shows clusters of nodes which are marked by overlaid color and are numbered from 1 to 17. These were discovered with the default setting of a standard community detection algorithm, Walktrap^14^ (see Methods). We observe that disease codes with the same starting alphabet are more likely to belong to the same cluster. The most prominent of these is cluster 3 (orange) most of whose codes begin with ‘O’ or ‘P’ and these correspond to pregnancy-related disorders. Slightly less pronounced examples include cluster 7 (light green) mainly of mental disorders (category F) and clusters 9 (dark green) and 2 (red) with predominantly circulatory system diseases (category I) and those leading to it.

We use network modularity (a metric that evaluates the effectiveness of a particular cluster decomposition of nodes on a given, fixed network) to compare the clusters obtained from the algorithm and those defined by the starting alphabet of the disease. Supplement Fig. 13 shows the curve of network modularity across different thresholds for the two cases. We find that, although the algorithmic detection of clusters led to greater modularity, the naive alphabet-based method scores fairly high as well (far greater than what would be expected by chance which is zero modularity). This is a more robust demonstration of the fact that diseases grouped under the same alphabet, and hence similar to each other as a biological/clinical category, cluster together^4^,^7^.

We argue that the greater proximity among diseases of the same category implies that patients having a specific disease are likely to be diagnosed down the line with another condition that affects the same organ or system, than something unrelated to it. For example, categories F3, F4 and F5 are all connected to one or the other form of mental/mood/behavioral disorders.

The presence of in-degree hubs in clusters suggest common symptoms of other related diseases. The largest and most distinctive such hub (in-degree 13, and out-degree 1) is ‘I1’ - hypertension and associated disorders, not unexpected, but this observation provides additional support for the validity of the metric. Likewise for the other in-degree hub ‘J1’ (in-degree 13, and out-degree 1), which comprises of pneumonia listed under different bacterial and viral causes, the edges from bacterial and viral diseases (A3, B2, B5) strengthens the putative causal claims in the subsequent analysis.

In much the same way, several sectors in the network are consistent with our biological and medical knowledge. We find it only reasonable that mood and behavioral disorders are related, and that pregnancy related complications would lead to complications during delivery and beyond. We can find even more powerful validation of the causal information encoded by the directed edges. For example, the directed path, *Q*0 → *G*9 → *I6* comprise diseases described as congenital malformations of the nervous system (Q0), disorders of the nervous system (G9) and cerebrovascular diseases (I6)^15^,^16^. While we ought to exercise caution in interpreting conditional dependence beyond a pair of diseases with our analysis (one would expect the edge *Q*0 → *I*6 too and that does not appear because, at CIF of 0.5, it falls below the threshold), the directionality of this triplet is remarkably consistent with our conception of disease development of the nervous system.

Thus the fourth step in our layered network analysis procedure is to turn to specific edges that we find interesting in Fig. 3 and then unpack the composite nodes that are linked by the edge. We constructed a bipartite network on these unpacked nodes (in three-digit precision) by drawing directed edges from nodes of the first set to the second based on CIF measure. We set a threshold and include interactions stronger than that. As this is a smaller network, there was no requirement for an upper bound.

**Figure 4:**
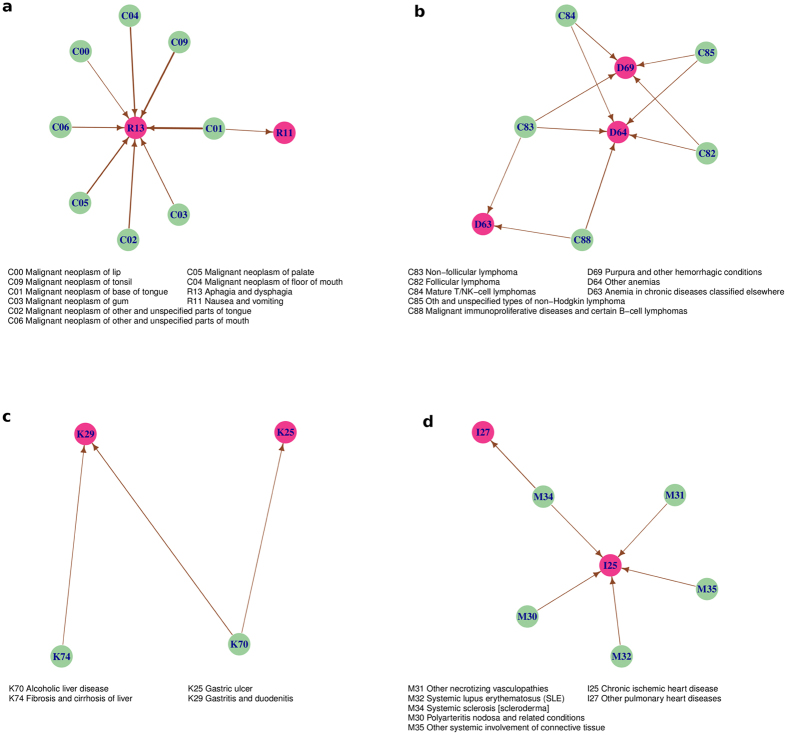
Expanded bipartite network for sets of four edges chosen from Fig. 3 that provides evidence towards validating our systematic procedure. The direction of links of the bipartite network is consistent with the original edge. (**a**) Mouth Cancer (C0) and digestive system problem (R1) (**b**) lymphomas (C8) and anemia/purprua (D6) (**c**) liver diseases (K7) and stomach ulcers (K2), (**d**) connective tissue disorders (M3) and heart diseases (I2).

As a validation of our approach, we unpacked a set of four edges (Fig. 4) that shows disease directionality that finds excellent agreement with existing medical knowledge. We color-coded the edges such that the subset of nodes corresponding to the composite node at the start (end) of an edge (in Fig. 3) appear green (dark-violet). We consider first the bipartite network between the composite nodes C0 (‘Cancers of tongue, mouth, lip etc’) and R1 (‘Symptoms of digestive problems’) and find that almost all of these cancers point to the node R13 (‘Asphagia and Dysphagia’ or difficulty swallowing), which of course is a very clear symptom. We next unpack the composite nodes C8 (‘Lymphomas’) and D6 (‘Anemia/Purpura/Coagulation Disorders’), and the resulting bipartite network shows that several types of lymphomas lead to anemia and purpura, and this progression is well-known^17^,^18^. The expanded subnetwork of K7 (‘Liver Diseases’) and K2 (‘Stomach/Duodenum Diseases’) shows that cirrhosis and alcohol related diseases of the liver are followed by gastric and duodenal ulcer, associations that has long been suspected, even if evidence has been mixed^19^,^20^,^21^. Next, we look into the subnetwork of M3 (‘Connective Tissue Disorders’) and I2 (‘Heart Disease’) and the progression of diseases such as systemic lupus, scleroderma and polyarteritis nodosa to pulmonary heart disorders is also well-known^22^,^23^.

### Novel Putative Disease Causal Patterns

**Figure 5:**
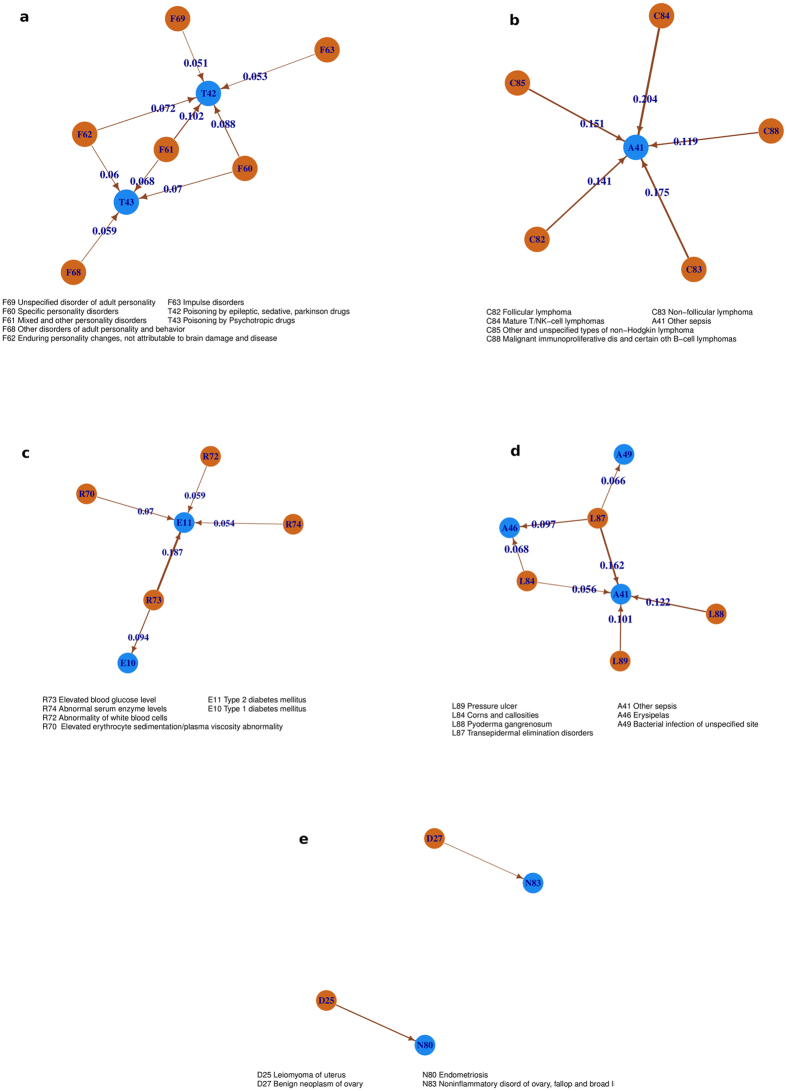
Bipartite network for various pairs of composite nodes that were deemed as representing significant new information about the constituent diseases and their relations. (**a**) mental disorder (F6) and poisoning by drugs (T4), (**b**) lymphoma (C8) with sepsis (A4), (**c**) anomalies in blood examination (R7) and diabetes (E1), (**d**) skin disorders (L8) to sepsis (A4), (**e**) benign Neoplasms of female genital organs (D2) to noninflammatory disorders of female genital tract (N8).

Figure 5 shows the unpacked, bipartite sub-network for four edges that we deemed interesting in the original network in Fig. 3. The directed edge (cluster 17 in Fig. 3) from ‘F6’ (disorders of adult personality and behavior) to ‘T4’ (different forms of poisoning, often by psychotic and hallucinogenic drugs) is expanded in Fig. 5a where nodes in light blue (red) represent diseases coded by ‘F6X’(‘T4X’). The direction of edges follow that of the original link in Fig. 3, namely, from ‘F6X’ and ‘T4X’. The thickness of the edges indicates the strength of the CIF measure. We believe that, this specific association is not well known or well studied in the literature on the subject.

The finer view offered by this subnetwork points to possible poisoning by prescription drugs of individuals diagnosed with personality disorders. The strong putative causal link (threshold 0.05, see Supplementary Fig. 12) between these conditions suggests that patients with mental disorders are far more likely to have a poisoning episode from the drugs than be diagnosed with any other disease. This may be explained by accidental poisoning using medications for the particular disorder^24^. Some of these may also represent suicide attempts as this fatalistic tendency is known to be prevalent among those diagnosed with mental disorders. There have only been a few studies that have explored this issue in the past^25^.

Next, we turn towards the association between lymphoma (C8) and sepsis (A4) (cluster 4 and 8 in Fig. 3) shown in Fig. 5b. This fact is not altogether unknown in medical/clinical studies, but certainly the severity of the association (CIF threshold 0.1) appears to be less understood. In particular, the disease code A41 (‘Other Sepsis’), mainly includes sepsis caused by *Staphylococcus aureus*, and the connection between lymphomas and this bacterium has not been addressed in literature to the best of our knowledge. We believe that this hypothesis is indeed true, it would contribute towards a better diagnosis and guide patients and doctors to be on a lookout for signs of sepsis once a diagnosis of lymph or blood cancer has been made.

Another interesting association, in cluster 2, is between R7 (abnormal findings in blood) and E1(diabetes). The expanded bipartite network in Fig. 5c shows that discovery of abnormalities in WBC and serum enzyme levels are putative causal priors to the onset of Type II diabetes. Abnormal serum enzyme levels as a predictor for diabetes have only been speculated earlier^26^ and our findings lend further support to the hypothesis. The same is true for WBC where we found only one large scale study suggesting that high WBC levels was a predictor for diabetes^27^. The idea of plasma viscosity as a risk factor for diabetes has also recently gained attention^28^. We believe that these putative causal associations warrant further investigation.

Finally, we consider the edge (cluster 8) from skin disorders (L8) to sepsis (A4). Figure 5d has a strong threshold of 0.05 and this bipartite subnetwork shows that pressure ulcers, callosities, pyoderma gangrenosum and pigmentation disorders has a higher chance of leading to sepsis. Once again, we believe that these are novel findings or at the very least, the degree of these associations were previously underestimated.

### Autoimmune Disorders and Cancer Synergies

Although there have been studies that have considered the possible connections between AI and cancer^29^,^30^,^31^ and examination of specific pairs of diseases^32^,^33^, there is still a lot that is not well understood. We explored new insights that this disease network reveals about the associations between autoimmune disorders (AI) and cancer.

As a test of the integrity of our data, Supplement Fig. 14 shows the comparison between the relative risk (RR) calculated from the cohort samples and the summary of results from different clinical studies reported in^34^. We find fair agreement between the two sources and this is only a crude check that our numbers are roughly compatible with previous studies and will not be used further in our analysis.

**Figure 6:**
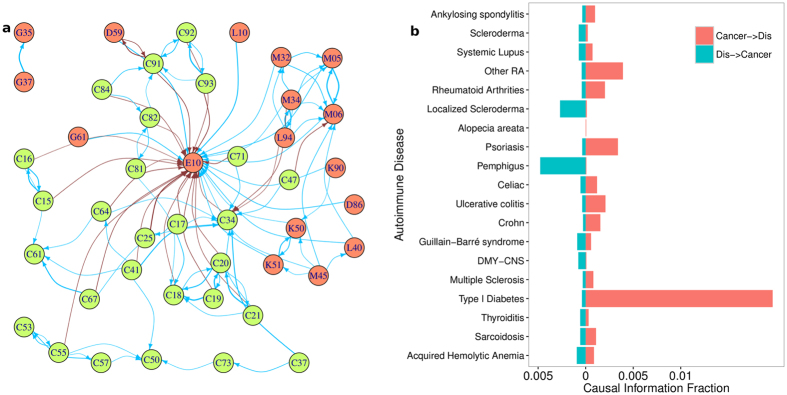
(**a**) Nodes in red(green) are generally AI(cancer), and blue (brown) edges connect diseases of same (different) category. The thickness of the edge indicates the strength of CIF measure. Threshold for an edge is 0.01, implying that at least one percent of patients go on develop the target disease. The ICD 10 code descriptions are - D86: Sarcoidosis; M45: Ankylosing spondylitis; D59: Acquired Hemolytic Anemia; E06: Thyroiditis; E10: Type I Diabetes; M34: Scleroderma; G35: Multiple Sclerosis; G37: DMY-CNS; K50: Crohn; K51: Ulcerative colitis; K90: Celiac; L10: Pemphigus; L40: Psoriasis; L63: Alopecia areata; M32: Systemic Lupus; M05: Rheumatoid Arthrities; M06: Other RA; L94: Localized Scleroderma; G61: Guillain-Barré syndrome; C15: Esophagus; C16: Stomach; C17: Small intestine; C18: Colon; C19: Recto-Sigmoid; C20: Rectum; C21: Anal; C25: Pancreas; C34: Lung Cancer; C37: Thymus; C41: Bone and Cartilege; C47: Peripheral Nerves and Nervous System; C50: Breast; C53: Cervix; C55: Uterus; C57: Ovarian Caner; C61: Prostate; C64: Kidney; C67: Bladder; C71: Brain; C73: Thyroid; C81: Hodgkin lymphoma; C82: Follicular Lympoma; C84: NK/T Cell lymphoma; C91: Lymphoid Leukemia; C92: Myeloid leukaemia; C93: Monocytic leukaemia (**b**) Associations between individual AI with all cancers, the bar plot heights corresponding to median values of CIF measure. In general, cancer precedes the disease in question, although there are exceptions.

To explore further the associations between AI and cancer, we focused on the subset of diseases considered in^34^ (see Methods Section for more detail) and constructed a directed network on them. We note here that we do not collapse the network as we did earlier because the number of nodes here is small enough that the structure of the network retains sufficient clarity with all the nodes. This is shown in Fig. 6a where the edges among AI nodes, cancer nodes, and those from one to the other are marked by different colors.

We observe that AI diseases are more likely to be clustered together than those of cancer (the cancer clusters are observed mostly among similar diseases - intestine-colon area C17-C18-C19-C20, leukemia C91-C92-C93). We also note that there is a greater likelihood for a secondary development of a different AI disease following an existing one than there is for cancer. For example, Celiac Disease (K90) is found to be a precursor to Crohn’s Disease (K50). The same is true for Systemic Lupus Erythematosus (M32) which can lead to rheumatoid arthritis (M05 and M06).

Figure 6b examines the overall causal relationship between cancers and a specific AI disease by showing the median of the CIF values from all cancers to the particular AI disease (red) and the other way around (green). We find that rheumatoid arthritis (RA) is preceded by cancer rather than the other way around, contrary to the claim made in the review^34^. The same is the case, at least on average, for Crohn’s Disease, Psoriasis and Type I diabetes.

On the other hand, there are other cases where the autoimmune dysfunction is observed before the onset of cancer. Those with Scleroderma, Localized Scleroderma and Pemphigus, had a higher risk of developing cancer than the other way around. And yet for others, such as Systemic Lupus Erythematosus (SLE) and Multiple Sclerosis, there is no clear direction in the order of conditional disease development.

### Focus on Specific AI Diseases

In line with our layered approach, we want to examine each of these AI diseases at a closer level, seeking to identify the specific cancers that are strongly associated with it. The subnetwork of interest, then, is the one induced on the vertex set consisting of the all cancer nodes and the particular AI in question. Ignoring cancer-cancer interactions we obtain the required graph that has the AI disease at the center, and outgoing and incoming causal associations with different cancers.

**Figure 7:**
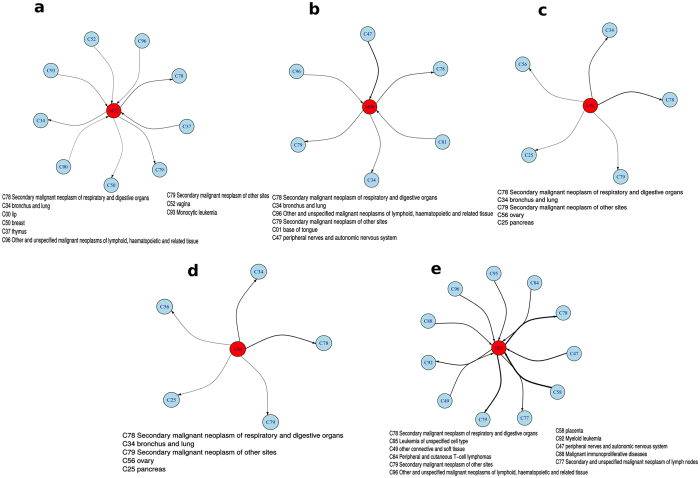
Significant associations of specific autoimmune diseases with cancer. Cancer associations of (**a**) Systemic Lupus Erythematosus (**b**) Rheumatoid Arthritis (**c**) Localized Scleroderma (**d**) Systemic Scleroderma (**e**) Aplastic Anemia.

Beginning with SLE (M32, Fig. 7a), we find that the disease is followed by breast cancer, an inference, if correct, appears to have eluded earlier studies^35^,^36^. In much the same way, while we find causal connection to Rheumatoid Arthritis (RA) in patients with peripheral nerves cancer, previous research has not reached any specific conclusion. Interestingly, lung cancer figures in the subnetwork for both cases, whereas kidney cancer does not (in the review^34^, though, kidney cancer is found to have a higher risk than lung cancer).

We find a more definitive causal structure in the case of disease associations of scleroderma and localized scleroderma (Fig. 7c,d). Despite the fact that several of these cancers have previously been associated^37^, this subnetwork clearly shows that scleroderma occurs (or at least is diagnosed) before malignancies of breasts or lungs^38^,^39^. The development of pancreatic and ovarian cancer that we find has not been reported earlier, to the best of our knowledge.

Turning to Aplastic Anemia (Fig. 7e), we find both strong and unambiguous associations. Apart from the secondary cancers (coded by C70’s), the other tumors of the blood, nerves and placenta all occur before the onset of the disease. Either this points to pathological associations that have previously remained unknown, or it suggests that cancer treatments including chemotherapy can lead to its development.

## Discussion

We have provided a comprehensive framework for analyzing the data on diseases for a large cohort of patients, involving examining its properties at multiple levels. We inferred the overall strong association between diseases from analysis of the comorbidities in the data-set. We further showed that the distribution of individuals with a certain number of diagnoses follows a power law, implying that the likelihood to be diagnosed with another disorder depends on the number of diagnoses already made.

Our key contribution is developing a systematic procedure for constructing directed networks on diseases and inferring significant new associations from it. Realizing that our network, much like all the others uncovered in this field^4^,^5^,^6^,^40^ is dense and clusters trivially among similar diseases, we collapsed similar nodes and constructed a network of composite disease states, limited the effect of trivial edges by setting an upper bound, and unpacked interesting edges into a bipartite network.

It is important to note that all the specific claims and descriptions of disease associations (Fig. 5) are based on 3 character disease codes, even though the selection of the subnetwork in which they are located is based on the collapsed, 2 character coded network. Thus we consider the collapsed network to offer a coarse-grained picture and a tentative understanding of disease relationships at a category level. This permits identification of possible candidate composite node pairs that may contain genuinely interesting and novel associations. In other words, the collapsed network is an intermediate step for more detailed analysis, and is therefore not recommended to directly make biological or clinical claims based on the associations found at this level of representation.

In contrast to previous work^6^ that looks only among co-occurrences to determine directionality of disease trajectories, our measure emphasizes the fraction of individuals with a given disease developing another. This is a critical difference in some cases: for a pair such as specific heart disease and a symptom such as hypertension, the CIF links the first to the second, even though among patients having both, the typical order of occurrence is the other way around. This is because the number of patients with hypertension is far greater than that for any specific heart disease, and hence there will be several instances of the former occurring before the latter that is merely an artifact of the disparity in prevalences (see Supplement Section H for more details on this and also specific examples where we get opposite directions). It should also be noted from the cumulative distribution (Supplementary Fig. 12) of CIF shows that even a value of 0.01 is stronger than 91% of all pairs. All these facts combine to increase our confidence in the causal nature of the edges in the networks we have shown.

Although we do not discuss it here, we constructed networks with other measures such as relative risk and *ϕ* correlation but these did not yield any new information. A discussion of other measures, their biases and their relation with CIF is given in Supplement Section G.

In contrast to previous work, our data-set included events from cause of death and disability pension instances and thus the associations inferred between diseases involving either are likely to be more reliable.

Although our final thresholds for the network in Fig. 3 were chosen by exploring different ranges, the claims about specific pairs of diseases is independent of this choice. It is important to realize that a different threshold would lead us to consider a different set of associations. Nonetheless, as we demonstrate in Supplement Section K.1, the selected communities and the degree distribution with respect to the thresholds are stable, thus ensuring that the structure identified by a particular threshold is not arbitrary. The heuristic considerations that guided us in the selection of thresholds chosen were (a) the network size and edge density remain moderate that the structure is clear and can be interpreted (in the range of 100 each, which determines the interval of the bounds) and (b) setting upper bound where interesting links begin to emerge.

However other interesting -especially local- structures and associations can be further uncovered in this network using the methodology we have prescribed. We’ve also observed that the most interesting links were identified through a more manual, rather than in an algorithmic, way, and additional work would presumably follow a similar procedure. Moreover, even the choice of our groupings could be chosen in a more nuanced fashion, paying closer attention to pathological characteristics of the disease codes. We, thus, argue that the limiting factor, then, is the sheer vastness of potential networks and the associated effort of manual detection. Likewise, there are other large disease categories for which a targeted exploration can be done using the approach we have taken for AI and cancers.

## Materials and Methods

### Data

The data for this paper was extracted from a composite database of information on all inpatient care, disability pension and cause of death in Sweden for a cohort defined as all residents in the country of age 16 to 64 at 31 December 1994 (N = 5,512,469). The ratio of men to women in the cohort was nearly 1:1. Moreover, at 1994, the distribution of patients across the age range was nearly uniform with the lower, middle and upper quartiles falling at 27, 39 and 50 years old (see Supplement Fig. 1). Data was obtained from high quality registers maintained by Statistics Sweden, the Swedish Board of Health and Welfare, and the Swedish Social Insurance Agency and was linked at an individual level and then anonymized^41^,^42^.

Data (date and all diagnoses) at individual level for all inpatient care, disability pensions and deaths occurring in the years 1997 through 2009 were extracted for the analysis. The data was encoded at 3 or more positions of precision. The extracted data has been downselected to entries conforming to the ICD-10^13^ coding standard (some other codes may be present in original data, but has been discarded at this step), with dates from 1997 through to 2009, translated into 3 position headings to keep the number of nodes in the graph reasonable. We note that earlier studies^4^,^5^,^6^,^7^ also used the same level of classification in constructing disease networks. Number of patients in this set is 2,901,783.

We would like to remark that all incidence rates and measures use the total cohort size N, and not the number of patients who have had at least one encounter. This is because we are following the entire cohort and frequencies and probabilities are calculated with respect to that.

A selection is made from the composite database and the diagnostic codes are extracted in time order. Duplicate codes are ignored, as we want to maintain time order. From the ordered list, disease pairs are generated in such a fashion that an example code list as (A,B,C,D) gives the disease pairs (AB,AC,AD,BC,BD,CD). Output is in the form of nodes (ICD-code, prevalence) and co-occurrences (Source ICD, Target ICD, Comorbidity count).

Further, while constructing the network of diseases, we eliminated those ICD codes from further analysis that merely describe events aside from development of disease (accidents, assaults, complications from surgery, contact with health service), codes that correspond to health status not related to disease (immunization information for example). This lead to excising codes starting with *V*,*X*,*Y* and *Z*. We also excluded diseases whose prevalence was less than 20 in order to minimize statistical errors.

### Null Distribution for Disease Frequency

The null distribution in this case is in fact the Poisson distribution with parameter 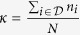, the average disease incidence, and 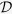 is the set of all diseases. Thus, the probability of an individual having *b* diseases is given by:


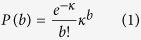


We argue that this is correct by demonstrating consistency with the help of combinatorial identities of disease co-occurrences and disease diagnoses rates. Let *N* and *N*_*v*_ be the total number of patients and the numbers that were diagnosed with exactly *v* diseases respectively. The following is always true:


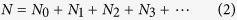



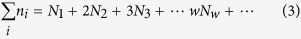



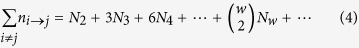


The first identity follows from the definition; the second by counting total prevalence in two ways - adding prevalence of every disease (left-hand side) or summing contributions to that total from each category of patients (right-hand side); the third, just as second, by counting differently - summing them directly (left-hand side) or the number of disease pairs an individual with *v* diseases would contribute to, i.e., 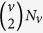.

On plugging Eq. (1) in the right hand side of combinatorial identities of Eq. (4):


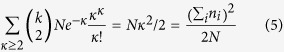


which is in fact what the left hand side would be if diseases were uncorrelated up to a term of order 1/N


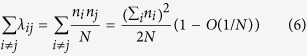


This provides an indirect, but strong support for our hypothesis that individual disease diagnoses follows a Poisson distribution.

### Normalized Frequency

In Supplement Fig. 2, the normalized occurrence on the y-axis is obtained by first calculating for every age *a*, the number of individuals *N*_*a*_ in the entire cohort who attain that age at some point in the entire time span over which the data was collected (1997–2009). To do this, we use the information on the distribution of ages of individuals in the cohort at Jan 1, 1997. The total number of diagnosis at age *a* is then divided by *N*_*a*_ to give the frequency of occurrence.

### Causal Information Fraction Measure

We wish to assign putative causal directions between a pair of disease *i* and *j* based on how often disease *j* follows *i* or the other way around. The null hypothesis of causal independence between two diseases is the equality of *n*_*i→j*_ and *n*_*j→i*_). However, rejecting the null hypothesis does not in any way measure the **strength** of causal association. What we are really interested in testing is finding out if the likelihood of developing disease *j* following *i*, i.e.,


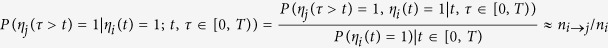


where *η*_*κ*_(*t*) is 1 or 0 depending on whether disease *κ* was diagnosed at time *t* or any time earlier. If this likelihood was the same as that of occurrence in the overall population, the ratio would be proportional to:


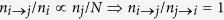


which gives us exactly our original null hypothesis. Thus, the null condition of the present test is identical to that of the previous case. However, assuming that there is a causal effect, then the fraction *n*_*i→j*_/*n*_*i*_ is a direct measure of how strong that effect is. Correcting for the null condition, we obtain:


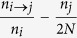


We call this the **Causal Information Fraction** (CIF). We also point out that a directional measure (rather similar to ours) was proposed in the original paper on disease networks^5^, but there is a fundamental problem in that approach which we describe in the Supplement Section H. We also comment on the method used for directions in^6^. Indeed, what we are using is a generalization of the conditional probability that was used in^4^ by including directions.

We note that, even when this measure of association is strong, it does not necessarily imply that one disease causes the other; it may well be that there are some joint causes to both that manifest earlier in one than the other. Although all of our analysis and results are based on this measure, we did construct networks with the other measures but they failed to reveal significant new interactions. Also, our data mainly concern more severe diseases, having led to hospitalization or disability pension or death. Patients might have been diagnosed as out-patient a long time ago.

Even though we constructed networks with the undirected measures, we obtained the most clear and interesting results by using the directional information. Thus, the networks and most of the analysis we have carried out here are based on this measure.

### Composite Network Measures

When diseases of the form ‘[alphabet]*αβ*’ is collapsed to a single category ‘[alphabet]*α*’, the statistics for the set of composite nodes are calculated by summing the prevalences and co-occurrences of the diseases within their respective composite node(s). For example, if we consider two composite nodes, 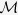 and 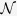, with constituent diseases 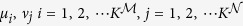 with prevalences 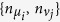, the prevalences for 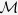 and 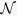 are defines as:


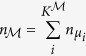



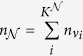


Likewise the pairwise comorbidity is defined as


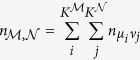



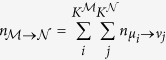


Every measure used before can be defined as before, except we use the comorbidities and prevalences values of the composite nodes. An astute reader will notice that there is a sleight of hand in the second summation because it over counts those individuals that have two or more distinct pairs of diseases from the composite pair. However, the error due to this is quite small and can be ignored.

### Unpacking Composite Node Pair

Given a directed link 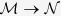 in the composite node network, we ‘unpack’ this by looking causal associations of the form 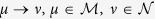, as determined by the CIF measure. It should be noted that, unlike the case of the composite node network, this is a strictly bipartite network, with only a single lower threshold, and the thickness of the edge represents strength of measure.

### Community Detection

We use the Walktrap algorithm^14^ to identify communities which is based on generating a Markov process for jumps from every node to every other node from the adjacency matrix. These transition probabilities are used to define a ‘Euclidean’ measure for distance between two nodes. A hierarchical clustering algorithm is then applied to the distance matrix resulting in a dendrogram. The choice of the cut in the dendrogram is done to maximize the modularity score.

In our work, we used the algorithm with the default settings (i.e., no edge weights and number of steps being 4) as is found in the R igraph package.

### AI Cancer Disease Selection

The AI subset followed the selection made in^34^ with the exception of those diseases that were encoded by greater precision than the three character codes. This elimination was done to avoid assigning prevalence of a three-character code to a disease that is specified at a four or five character precision level. We included in our subset those cancers that were listed in Table II in^34^ along with the hematologic cancers listed in Table III of the same review.

## Additional Information

**How to cite this article**: Kannan, V. *et al*. Conditional Disease Development extracted from Longitudinal Health Care Cohort Data using Layered Network Construction. *Sci. Rep*. **6**, 26170; doi: 10.1038/srep26170 (2016).

## Supplementary Material

Supplementary Information
