# Supplementary material for: Conditional Disease Development extracted from Longitudinal Health Care Cohort Data using Layered Network Construction
Source: Sci Rep. 2016 May 23;6:26170. doi: 10.1038/srep26170 (PMC4876508; doi:10.1038/srep26170)
Supplement: Supplementary Information [file srep26170-s1.pdf]

Conditional Disease Development extracted from Longitudinal Health Care Cohort Data using Layered Network Construction
Supplementary Material

Venkateshan Kannan[1,2], Fredrik Swartz[1,2,5], Narsis A. Kiani[1,2], Gilad Silberberg[1,2], Giorgos Tsipras[1,2], David Gomez-Cabrero[1,2], Kristina Alexanderson[5], and Jesper Tegner[1,2,3,4]

[1]Computational Medicine Unit, Department of Medicine, Solna, Karolinska Institutet, SE-17176,Stockholm, Sweden
[2]Center for Molecular Medicine, L8:05, SE-17176, Stockholm, Karolinska Institutet, Sweden
[3]Unit of Clinical Epidemiology, Department of Medicine, Karolinska University Hospital L8, SE-17176, Stockholm, Sweden
[4]Science for Life Laboratory, Stockholm, Sweden
[5]Division of Insurance Medicine, Department of Clinical Neuroscience, Karolinska Institutet, SE-17177 Stockholm, Sweden

A Poisson Distribution of Co-occurrences

Here we show that distribution of co-occurrences for a given pair of diseases with specified incidence rates under the null hypothesis that the two diseases are completely independent, is in fact, Poisson.

Concretely, if N is total population of the cohort, and n_i represents the number of people with disease i ($i = 1, 2, \cdots M$), and if all diseases are uncorrelated with each other and occur randomly in the population, the total number of ways in which we can have an overlap of n_{ij} individuals carrying both diseases i and j is given by:

$$\begin{aligned} C(n_i, n_j, n_{ij}, N) &= \binom{N}{n_i}\binom{n_i}{n_{ij}}\binom{N-n_i}{n_j-n_{ij}} \\ &= \frac{N!}{(n_i-n_{ij})!(n_j-n_{ij})!n_{ij}!(N+n_{ij}-n_i-n_j)!} \end{aligned} \tag{1}$$

where the first factor corresponds to choosing n_i elements (first set) from N, the second, the intersecting n_{ij} elements among the n_i, and the third, the $n_j - n_{ij}$ elements (second set - overlapping elements) from the remaining $N - n_i$ elements. Taking the log on both sides and using the Sterling approximation $\log N! = N \log N - N$,

$$\log C(n_i, n_j, n_{ij}, N) = N \log N - n_i(1 - \frac{n_{ij}}{n_i}) \log n_i(1 - \frac{n_{ij}}{n_i}) - n_j(1 - \frac{n_{ij}}{n_j}) \log n_j(1 - \frac{n_{ij}}{n_j}) \\ - n_{ij} \log n_{ij} - (N - n_i - n_j)(1 + \frac{n_{ij}}{N - n_i - n_j}) \log (N - n_i - n_j)(1 + \frac{n_{ij}}{N - n_i - n_j}) \tag{2}$$

Simplifying and retaining only terms that are first order in n_{ij} or higher:

$$\log C(n_i, n_j, n_{ij}, N) = \log C_0 + n_{ij}(1 + \log \frac{n_i n_j}{N - n_i - n_j} - \log n_{ij}) \tag{3}$$

Exponentiating, and using the Sterling approximation in reverse, we get:

$$C = \frac{C_0}{n_{ij}!} \left(\frac{n_i n_j}{N - n_i - n_j} \right)^{n_{ij}} \tag{4}$$

which is nothing but the Poisson distribution with (average $\mathbf{E}(n_{ij}) = \lambda = \frac{n_i n_j}{N - n_i - n_j}$ and the constant C_0 would be normalized to $e^{-\lambda}$.

This is of course what we would expect from naive considerations of probability theory. Thus one can calculate p-values from that of the Poisson distribution the p-value corresponding to an observation n_{ij}, then comes from the cumulative distributive function of Poisson :

$$F(n_{ij}) = \sum_{k \geq n_{ij}} P_{Poiss}(n_{ij}, \lambda) \tag{5}$$

B Symmetric Definition of Relative Risk

We note that the standard definition of relative risk considers rates of disease cases among sets exposed and not exposed to a given condition. Namely, for the 2×2 contingency table between a pair of diseases A and B: The relative

	A	$\bar{A}$	
B	p	q	$n_B = p + q$
$\bar{B}$	r	s	$n_{\bar{B}} = r + s$
	$n_A = p + r$	$n_B = q + s$	$N = p + q + r + s$

Table 1: Contingency table for a pair of diseases in a cohort

risk for disease A, in the presence of B is given by the ratio of occurrence of A among those having disease B to that occurring among those who do not have disease B. :

$$RR_{A|B} = \frac{p/(p+q)}{r/(r+s)} = \frac{p/n_B}{r/n_{\bar{B}}} \tag{6}$$

and likewise, swapping A and B

$$RR_{B|A} = \frac{p/(p+r)}{q/(q+s)}$$

Clearly, the metric is asymmetric with respect to the which disease is regarded as the exposure condition. Nonetheless, in our dataset the individual disease prevalence as a fraction of the cohort is very small, $p/s, q/s, r/s << 1$. Further, under the assumption that $p/r, p/q << 1$, which effectively implies that prevalences within the subset of patients having another disease to also be very small, it is easy to show that both of the above expressions for relative risk simplify to an identical form.

$$\begin{aligned} RR_{A|B} &= \frac{p/n_B}{r/n_{\bar{B}}} \\ &= \frac{pn_{\bar{B}}}{n_B r} \\ &\sim \frac{pN}{n_A n_B} \end{aligned}$$

This is quite well-known [2] and indeed, this expression has been used on similar cohorts which earlier studies on disease networks have considered [1].

C Age and Gender

Fig. 1 shows the cumulative distribution of the ages of individuals in the cohort at 1994. As can be clearly seen, the near-linear curve suggests that the density distribution is close to uniform across the ages 16-65.

In Fig. 2, we show a plot the variation of rate of diagnoses with age in our cohort for the entire set and men and women separately. We find that the number of diagnoses in general increases with age with the exception of the bump around 30 years that can be attributed to pregnancy-related problems.
To analyze the effects of gender, we only focused on those diseases that are present in both the genders. Fig. 3 shows gender-difference on the y-axis for every disease, given by $n_A^m/N^m - n_A^f/N^f$, where $n_A^{m/f}$ refers to the number of men and women with disease A, and $N^{m/f}$ is the total number of men and women in the population. The outliers (Tables 3 and 4) are once again quite expected - burn injury (T26, T23, T20, T31, T25, T22, T29, T30, T27), crushing injury (S97, S67, S57, S38, S77, S87, S07), cardiovascular diseases (I25, I40), Type II diabetes (E11), and cancers affecting the mouth, nose and throat

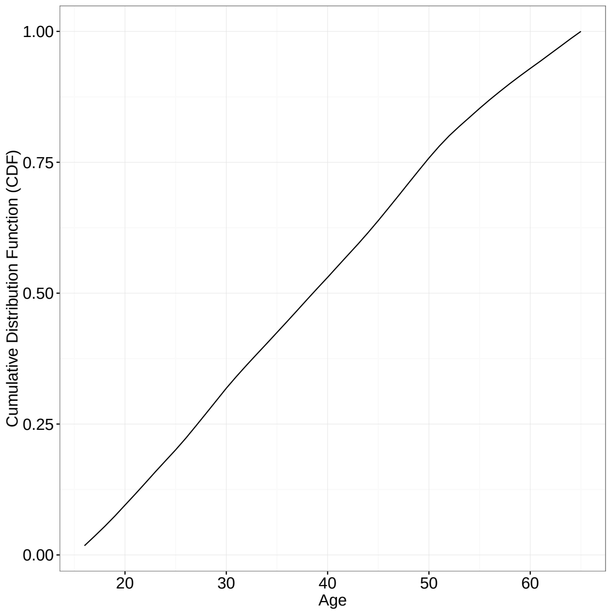

Figure 1: Cumulative distribution of the age of individuals in the cohort.

such as larynx (C32), sinus (C12), esophagus (C15) and tonsil (C09) occur more in men than in women. The last is most likely the result of tobacco use or perhaps occupational hazards of the construction industry that is typically dominated by men. Likewise, Osteoporosis (M81), thyroid problems (E01, E02, E03, E06, E21), eating disorders (F50), lactose intolerance (E73)and disorders of the cartilage and connective tissue (M35) are more prevalent in women, which is in accordance with existing knowledge.

Next, we look at a set of 30 autoimmune diseases (Fig. 4) to check for agreement with the well known fact that women are more likely to acquire AI diseases than men [3]. The higher proportion among women can be easily observed across different AI and indeed the overall ratio of prevalence between women and men is close to 2. In particular systemic lupus erythematosus (M32), rheumatoid arthritis (M05, M06) and thyroiditis (E06) affect women considerably more often than men.

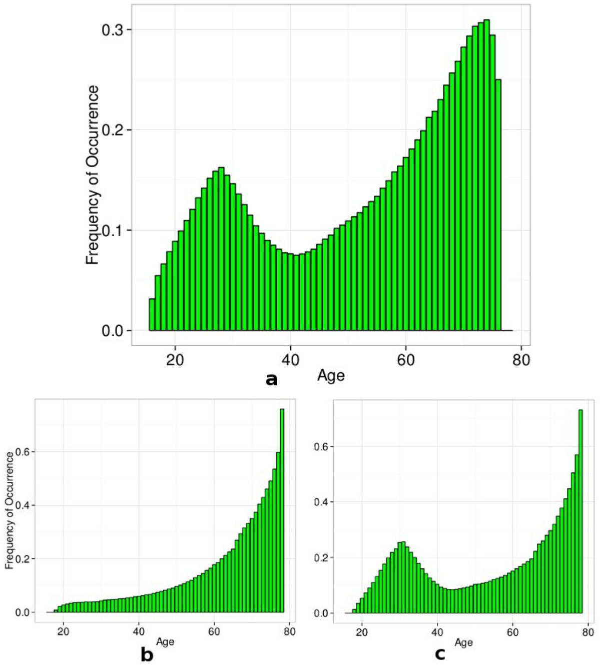

Figure 2: Variation of regularity of new diagnoses with age for the (a) entire cohort, (b) men and (c) women only. Note the peak that we see around age 30 that is present only in women and is related to pregnancy.

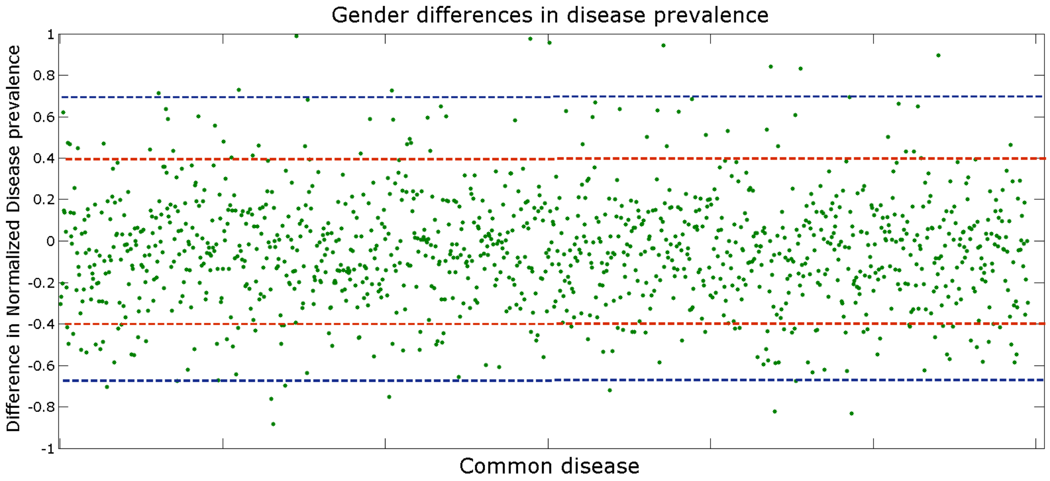

Figure 3: Plot of difference of prevalence between genders (normalized to the total number of each gender) for all diseases that commonly occur among both. The red and the blue dashed lines represent two thresholds for male (and female) dominated diseases.

D Networks with Higher Average Degree

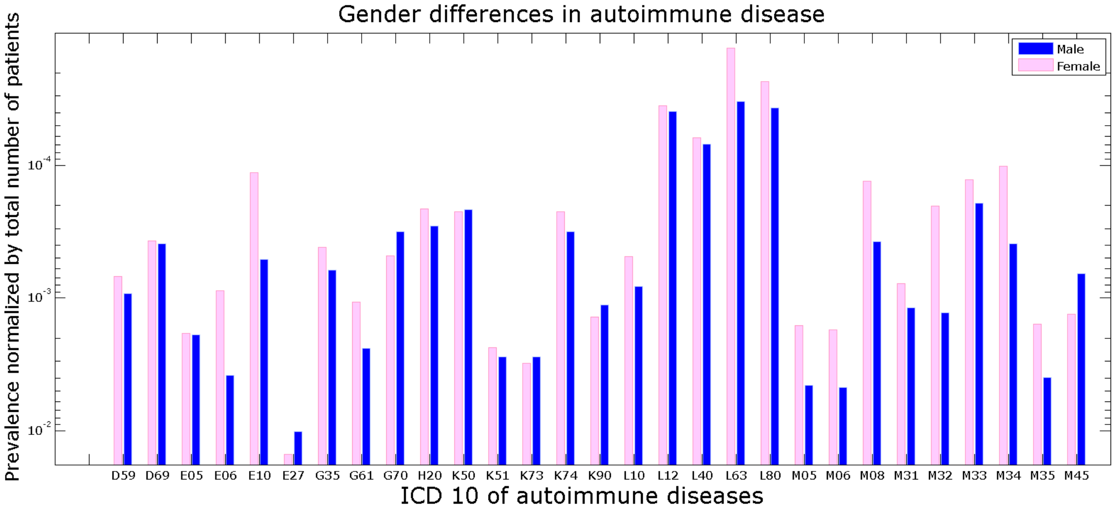

Figure 4: Comparison of prevalence of autoimmune disease among men and women, normalized to the total population (of each gender) in the cohort. List of AI diseases - D59: Acquired hemolytic anemia; D69: Purpura and other hemorrhagic conditions; E05: Thyrotoxicosis; E06: Thyroiditis; E10: Type 1 diabetes mellitus; E27: Other disorders of adrenal gland; G35: Multiple sclerosis; G61: Inflammatory polyneuropathy; G70: Myasthenia gravis; H20: Iridocyclitis; K50: Crohns disease; K51: Ulcerative colitis; K73: Chronic hepatitis; K74: Fibrosis and cirrhosis of liver; K90: Intestinal malabsorption; L10: Pemphigus; L12: Pemphigoid; L40: Psoriasis; L63: Alopecia areata; L80: Vitiligo; M05: Rheumatoid arthritis; M06: Other rheumatoid arthritis; M08: Juvenile arthritis; M31: Other necrotizing vasculopathies; M32: Systemic lupus erythematosus; M33: Dermatopolymyositis; M34: Systemic sclerosis; M35: Other systemic involvement of connective tissue; M45: Ankylosing spondylitis

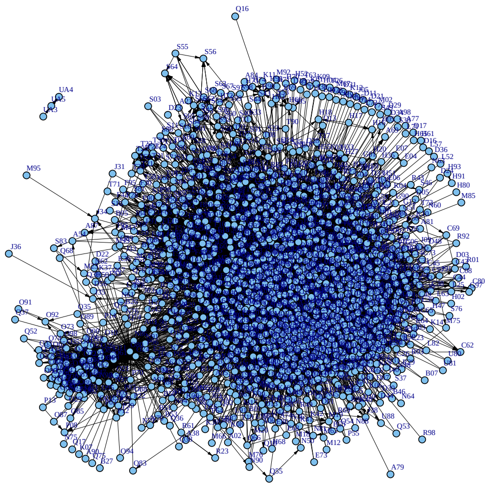

Figure 5: Full Network-Average Degree 20: Dense Interconnected network.

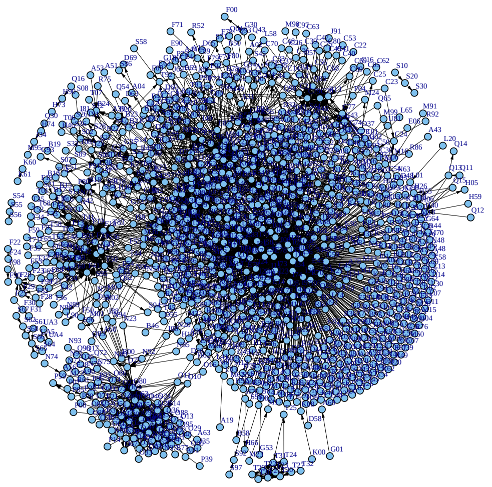

Figure 6: Full Network-Average Degree 5 :Still a giant cluster with criss-crossing edges.

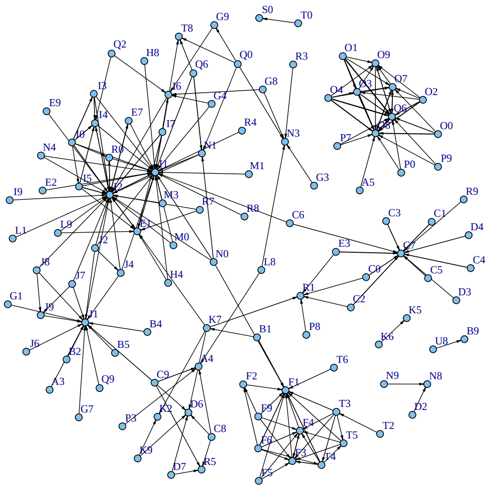

Figure 7: Network with the collapsed networks.

E Standard Network Tools

While direct application of network statistics and properties can lead to detection of interesting features in general, there are several reasons why the particular network constructed with disease comorbidites does not yield to such straightforward approaches.

To illustrate this, we consider the network constructed with the causal information metric and thresholds chosen such that it represents the strongest 2% of all interactions. The resulting network has 1400+ nodes and 44000+ edges. As we saw in the previous section, plotting this would just be a dense, overlapping layers of nodes and edges.

Turning to specific methods, the top 5 nodes with the highest degrees are Hypertension (I10), Other urinary system disorders (N39), Unknown source of Pneumonia (J18), Type II Diabetes (E11) and Complications of procedure (T81). More than 95% of the edges are in-coming for all the above, and this is to be expected given the specification of the disease (maybe except Type II Diabetes). What makes this of little use is because of the five, three refers to some unknown character or unspecified origin, and the most dominant, hypertension is merely a common symptom. This does not imply that all hubs are necessarily uninteresting, except that careful examination and additional knowledge is necessary to establish their significance (or not). Indeed, we do describe network properties, such as the communities and edge density, when we ultimately construct our network in Fig 3 (main text), but that follows pre-processing steps accompanied by adequate qualifications.

Likewise, consider the global clustering coefficient 0.14, which is far greater than the 0.02 that would be expected had the network been a random graph with the same average degree. Again, this demonstrates that diseases in the network cluster immensely, but this follows naturally from Fig 2 (main text) showing show the relative risk is distributed.

The diseases with highest vertex between-ness centrality are Chronic kidney disease (N18), Heart failure (I50), Type I Diabetes (E10) and Cytomegaloviral Disease (B25). It is however not immediately clear what the significance here is aside from the fact that these are also nodes with very high degrees.

As we have noted, an even bigger issue plaguing these statistics is that they are driven by the strongest links, which as we have noticed is trivial. We need to prune them systematically in order to infer something of value.

F Inferences from the Strongest Interactions

Here give examples of the strongest associations (CIF) from the collapsed network.We consider the edges with the highest weights:
O1 $\rightarrow$ O6, O4 $\rightarrow$ O6, O1 $\rightarrow$ O8, O4 $\rightarrow$ O8, O6 $\rightarrow$ O8, O0 $\rightarrow$ O8, O2 $\rightarrow$ O8 and O3 $\rightarrow$ O8, E3 $\rightarrow$ C7, C1 $\rightarrow$ C7, O2 $\rightarrow$ O6, O3 $\rightarrow$ O6, I5 $\rightarrow$ I2, C3 $\rightarrow$ C7 and

Description of ICD codes of strongest causal connections in the network	
O10/O14	**Hypertension pre-existing (gestational)**
O43/O44/O45	**Various placental disorders**
O60/O61	**Preterm labour and delivery**
O80	**Single spontaneous delivery**
C1/C2/C3	**Neoplasms of the respiratory and digestive organs**
C78	**Secondary neoplasms of respiratory and digestive organs**
E34	**Endocrine disorders (Carcinoid Syndrome)**
I5	**Heart failure and ill-defined heart disorders**
I2	**Heart diseases**

Table 2

C2 $\rightarrow$ C7.
Although these represent the strongest causal links, they are, at the same time, the most obvious and least interesting associations as well (see Table. 2). To keep out these out, we take the **counterintuitive step of introducing an upper bound on the CIF measure** which eliminates edges whose measure is greater than that value.
Although these represent the strongest causal links, they are, at the same time, the most obvious and least interesting associations as well. To keep out these out, we take the **counterintuitive step of introducing an upper bound on the CIF measure** which eliminates edges whose measure is greater than that value.

G Bias of Standard Measures

We observed that both the standard measures used in earlier literature, relative risk (RR) and the Φ correlation metric, have systematic biases, especially the former one. These have been reported before, [4, 1], and we explore the consequences of that bias in our dataset. We find that RR often inflates the degree of association between diseases that have smaller incidence rates. In order to observe this, we take all pairs of diseases, bin them by their expected number of co-occurrences, i.e $\frac{n_{12}N}{n_1 n_2}$, and consider the distribution of RR within each. This is shown in figure 8 , where it is very clear that RR values are exaggerated for disease pairs that have smaller expected co-occurrences. Indeed, when expected co-occurrence α is much (few orders) smaller than unity, then RR takes values that of order $1/\alpha$.

We can also understand the instability of the measure to small perturbations in the co-occurrences, by considering the partial derivative of RR(n_1, n_2, n_{12}) wrt n_{12}

$$\frac{\partial RR}{\partial n_{12}} = 1/\frac{n_1 n_2}{N} = \frac{1}{\alpha}$$

Again we find that small co-occurrences lead to wide variation in RR when the

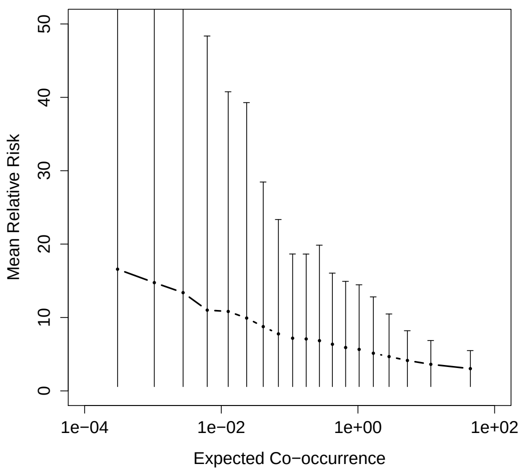

Figure 8: Bias in relative risk as seen in its dependence on the expected co-occurrence. The bars are the standard deviation of the RR scores for cases that fall within that interval. Expected co-occurrences are bunched together in bins of size $\sim$ 30000

expected co-occurrence is small. It should be noted that the dependence of the standard deviation of RR on the expected co-occurrence α assuming Poisson distribution for co-occurrences (i.e, no association), goes as $\frac{\text{Std. Dev. of Poisson with average}\alpha}{\alpha} = \frac{1}{\sqrt{\alpha}}$. For a pair of diseases, whose overall incidence is held fixed $n_1 + n_2 = C$, RR is better performing when the two incidence frequencies are more similar to each other. We will come back to this later.

Turning to correlation ϕ, we find that, whenever the incidences are widely separated in scales, the measure has a bias. From the definition, for prevalences n_1 and n_2 (and assuming $n_1 < n_2$)

$$\begin{aligned}
\phi(1,2) &= \frac{n_{12}/N - (n_1/N)(n_2/N)}{\sqrt{(n_1/N)(n_2/N)(1-n_1/N)(1-n_2/N)}} \\
&<= \frac{\min\{n_1, n_2\}/N - (n_1/N)(n_2/N)}{\sqrt{(n_1/N)(n_2/N)(1-n_1/N)(1-n_2/N)}} \\
&= \frac{(n_1/N)(1-n_2/N)}{\sqrt{(n_1/N)(n_2/N)(1-n_1/N)(1-n_2/N)}} \\
&= \sqrt{\frac{n_1}{n_2}\frac{1-n_2/N}{1-n_1/N}} \\
&< \sqrt{\frac{n_1}{n_2}}
\end{aligned}$$

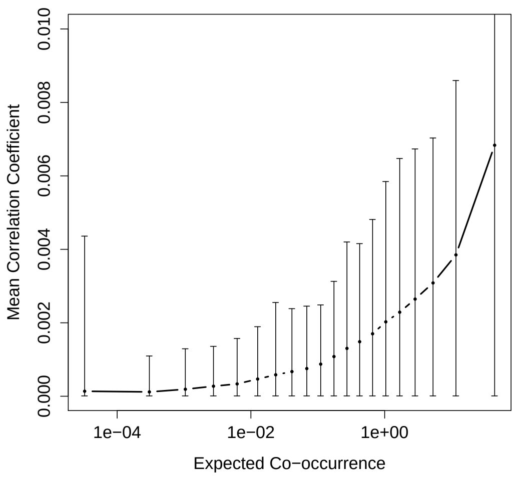

Figure 9: Mean and standard deviation of Phi-Correlation as a function of expected co-occurrence $n_i n_j/N$. Expected co-occurrences are bunched together in bins of size $\sim$ 30000

we arrive at an upper bound that depends on the ratio of the two prevalences, i.e, even if the two diseases are perfectly associated the ϕ measure will not be unity. Once again, we plot the variation of the ϕ for sets of disease pairs separated into distinct bins according to their expected co-occurrences. This distribution is shown in Fig. (9) and we find a similar bias as RR, except it is in the opposite direction, i.e., pairs with smaller expected co-occurrences have lower mean values of the correlation and the other way around for larger expected co-occurrences. We propose a correction that follows from the original expression and define the modified correlation ϕ^M :

$$\phi^{(M)}(i,j) = \frac{n_{ij}/N - (n_i/N)(n_j/N)}{min\{n_i, n_j\}/N}.$$

We follow the same procedure for plotting the potential bias as before, and find that our new measure performs significantly better.

We look into the consistency of these different measures by considering the correlation between the node degrees of the two networks that are obtained by setting thresholds for the edges based on a pair of measures. As the range of these measures is in general different and incompatible, our threshold is set based on the desired average degree of the resulting network. In Fig. 11, we show a heatmap of the correlation scores between the different networks obtained by this procedure with the average degree being between 10 and 50. As we suspected, relative risk (RR) based networks distinctly stand out compared to the others, all of which have a reasonable amount of overlap for each other. For the case of the causal-information fraction, we used the total degree (in- and out) when correlations were calculated.

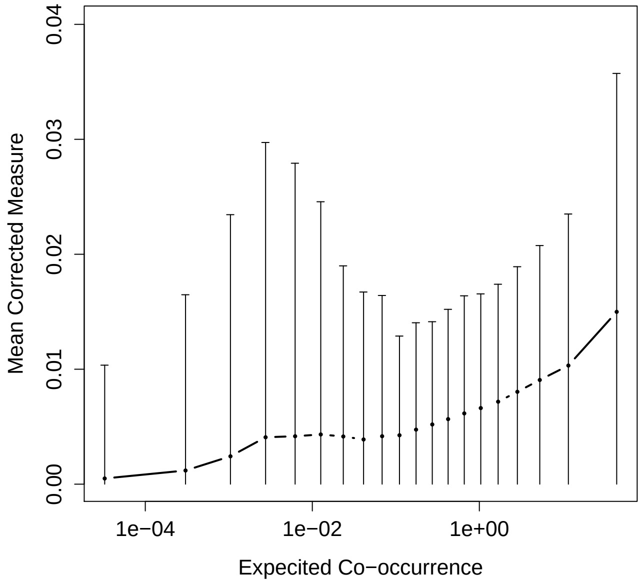

Figure 10: Mean and variation of corrected measure as a function of expected co-occurrence $n_i n_j / N$. Expected co-occurrences are bunched together in bins of size ~ 30000

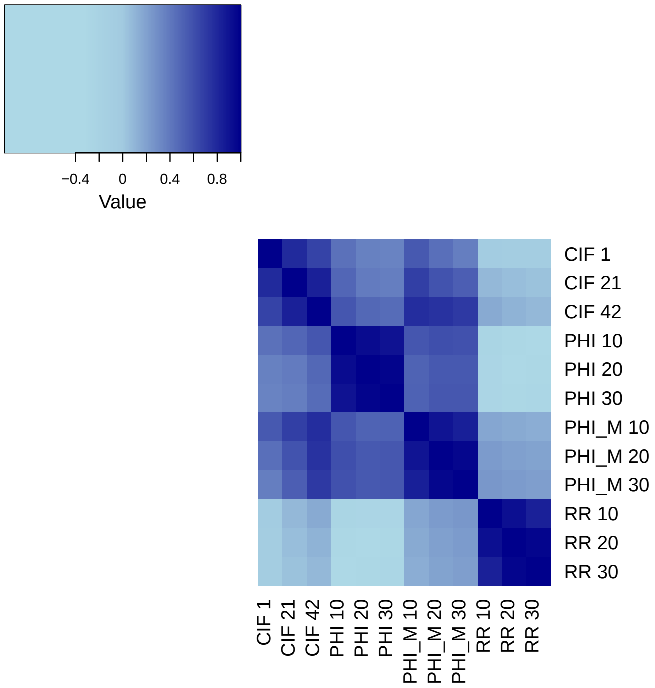

Figure 11: Correlation between the node degrees of networks with the numbers next to the measures indicating the average degree of the resulting network. Clearly, the network constructed with the relative risk (RR) measure is the most isolated among all the other networks.

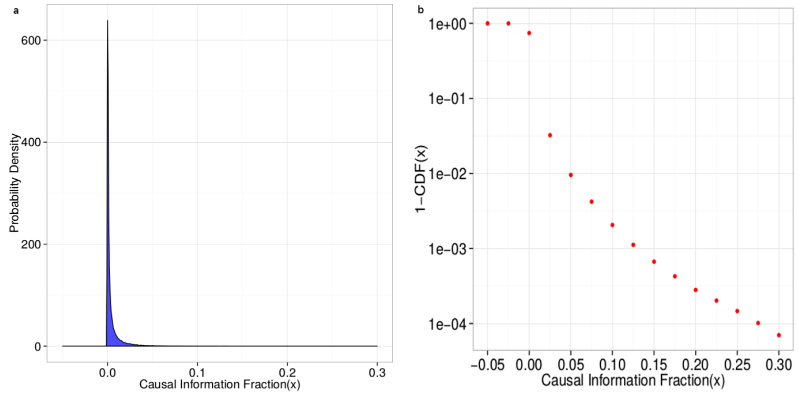

Figure 12: (a) Probability density and (b) Cumulative Distribution of CIF scores in the cohort dataset. The y-axis in subfigure (b) is 1-CDF, i.e., the fraction of pairs for which CIF is greater than the corresponding value on the x-axis. The plot clearly shows how rapidly this fraction drops as we go from 0 and enter $O(10^{-2})$. Specifically, note that even for a measure strength of 0.05, this fraction is less than 1%.

H Directional Measure

In Fig. 12, we plot the cumulative distribution function of the new metric, Causal Information Fraction (CIF). The set of values are from all pairs of diseases that was finally included in our dataset (after filtering out low prevalences and removing specific disease codes as mentioned earlier) for which there is at least one case of co-occurrence. As this leaves out pairs with no co-occurrences, the calculated value of CIF is underestimated for a given threshold. This gives a measure of how strong the associations we consider in the main text are.

We wish to compare our directional measure with the others that were proposed for similar pehnotypic networks in earlier works [1, 5]. In [1], they define the following metric for a pair of diseases A and B:

$$\Lambda_{A\to B} = \log_{10} \frac{n_{A\to B}/n_A}{n_{B\to A}/n_B}$$

However, as we have argued in the main text (see Methods), the null hypothesis of no causal association leads to the equality $n_{A\to B} = n_{B\to A} = n_{AB}/2$, and thus, $\Lambda^0_{A\to B} = \frac{n_B}{n_A}$, instead of being 1 as would be expected, could be arbitrarily high depending on the incidences of the two diseases. This is thus an unsatisfactory measure for all pairs whose prevalences are unequal which is the most generic case of our dataset.

In [5], they calculate the likelihood of obtaining the two numbers, $n_{A\to B}$ and $n_{B\to A}$ from the total n_{AB}, assuming that under null hypothesis the order of occurrence is evenly split. While the condition of the null hypothesis is indeed correct, the rejection of the null hypothesis does not lend good support for the alternate hypothesis that one disease likely causes the other. To understand this better let us assume the cohort

has a million patients (M) and also the fact that A is more likely to be a cause of B with about 5% of individuals with A go on develop B. Furthermore, through indirect effects B causes A but much less likely in that 1% of B patients develop A later. This would give $n_{A \to B} = 0.05 * n_A + w_1$ and $n_{B \to A} = 0.01 * n_B + w_2$, where w_i represent the numbers coming from random overlap which is $\sim \frac{n_A n_B}{N}$. Now, the proposed method would give a p-value for $A \to B$ that depends strongly on the relative values of n_A and n_B, and indeed when $n_B > 5n_A$, it is more likely to support the reverse directional association.

It might seem, superficially, that we are arguing against the two approaches ([1] and [5]) in a way that promotes the other. This is because of a very subtle fact that, under no association, the numbers in either direction are the same **regardless** of the incidence ratios, whereas when there is a causal association, the relative value of the occurrences in the two directions is an inadequate parameter to determine the strength of causal connection.

Moreover, even assuming no causation from B to A, this methodology would give significance even for very weak associations from A to B as long as n_A is sufficiently large. In other words, this does not measure the strength of the effect of causation, which is the more decisive indicator here.

More specifically, we consider some of the directions reported in just one figure of [5] that we find rather doubtful and compare with what we have using our method.

(a) Type II Diabetes (E11) $\longrightarrow$ Type I Diabetes (E10)
Their cluster shows the above edge from Type II to Type I, which is very unusual considering that Type I usually occurs earlier. Our measure indicates the putative causation to be the other way around(as would be expected).

(b) Hypoglycemia (E16) $\longrightarrow$ Nondiabetic Hypoglycemia (E15) This direction was found in [5] is particularly strange because hypoglycemia is usually caused by insulin administered to diabetes patients, and for hypoglycemia patients to be later diagnosed with its nondiabetic version is very odd. Our measure suggests the putative causation to be much stronger in the other direction, namely from E15 to E16.

(c) Angina Pectoris(I25)$\longrightarrow$ Heart Failure (I50)

Although the expectation would be that Angina Pectoris (chest pain) is a symptom of heart failure, but that does not appear to be so in the analysis of [5]. With our measure, we find that the putative causal association is stronger for I50 –¿ I25. This is a perfect example where, although examination of co-occurrences alone would suggest that chest pain precedes heart failure (a sensible feature, given that the symptom would appear before the disease), the likely cause and effect is reversed.

I Network Modularity

Given a network G, the node and edge set V and E respectively, and a clustering of the nodes into communities, $C : V \to \mathcal{C}$, we define the modularity as

$$M = \frac{1}{\sum_k d_k} \sum_{ij} \{E_{ij} - \frac{d_i d_j}{\sum_k d_k}\} \delta(C(i), C(j))$$

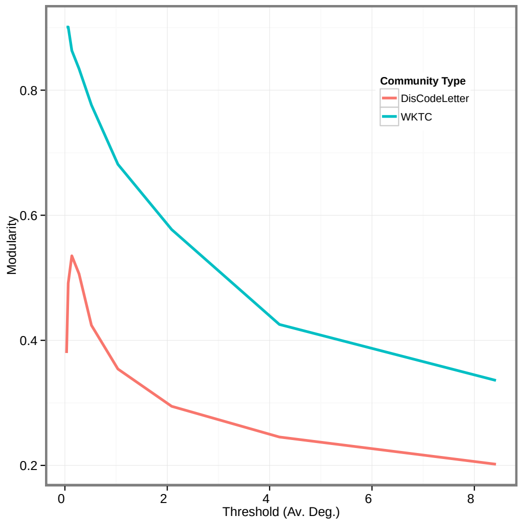

Figure 13: Comparing modularity scores between groupings based on disease coding and those discovered by algorithm. The network comprises of vertices that are two-character ICD 10 disorders and the grouping based on coding uses only the first alphabet. In general, for both curves, modularity increases as we make the threshold more stringent, indicating that network with strongest associations cluster more neatly. The exception to that is the kink we observe in the curve for the disease code-based modularity which suggests that, at the very highest thresholds, there is significant prevalence of inter-cluster edges.

which calculates the difference between the fraction of edges within a cluster and the expected number assuming that nodes are joined at random. We compare the modularity scores on the network shown in the main text (Fig 3) between the clusters (a) assigned through ICD 10 classification and that (b) obtained from the community detection algorithm (Fig. 13). In calculating the modularity here, the directionality of the edges was ignored.

J Comparison of Association from Study and Cohort

A comparison of the the relative risk measures between the median values of the study [6] and from the cohort is shown in Fig. 14.The reasonable agreement between the two of them is only meant to indicate that our data from cohort is reliable.

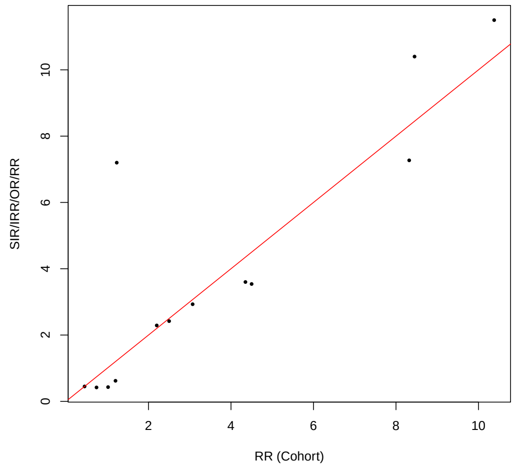

Figure 14: Comparing relative risk as measured from the cohort and the values obtained from the review [6] for specific set of diseases.

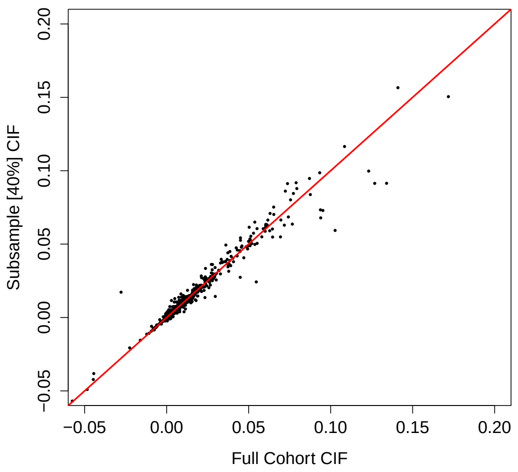

Figure 15: CIF values for 1000 randomly selected pairs of composite categories for the full dataset and that obtained from the subsampling. The red line represents the equation x=y.

K Network Stability

To test the robustness of the network constructed with respect to some anomalies and artifacts in the cohort data, we generated a separate dataset of prevalence and co-occurrences by randomly subsampling about 40% of individuals from the cohort of 5.5 million.

The stability of the network is fundamentally dependent on the robustness of the CIF measure that defines it. We therefore compare the CIF values for a set of 1000 randomly generated pairs of disease categories (Fig 15). We find excellent agreement between the two datasets.

We apply the more stringent criterion of overlapping edge fraction to directly compare networks. To do this, we generated 25 different subsamples of the original cohort, each with 1 million patients, or about 18% of the full cohort. In the first case, the networks constructed from the full and subsampled data have the same lower bound as expressed through average network degree. Fig. 16 shows the distribution of the fraction of overlapping edges for different lower bounds and we find that even for a narrow bound like 2, the overlap is about 68%.

Next, we starting with the network G on the full dataset for an absolute interval for the bounds (0.15,0.20), we constructed networks from the subsampled sets on a range of intervals, starting from the that of G and expanding in both directions. Fig 17 shows the distribution of the overlap fraction of edges as the interval widens, and we find that even as the interval expands to just (0.13,0.22) we have a three-quarters overlap.

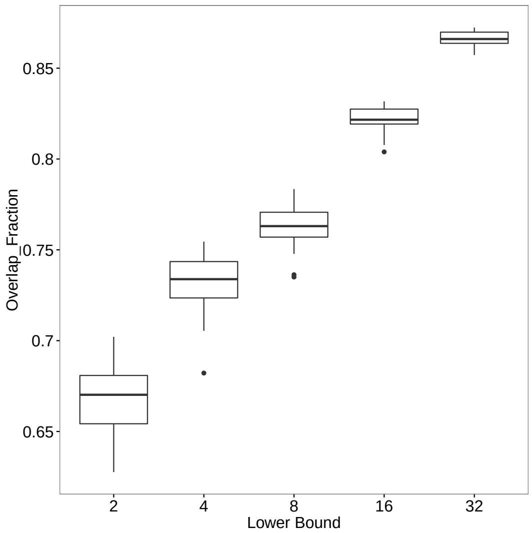

Figure 16: Boxplot of the distribution of overlap in directed edges between the networks from the full and the subsampled dataset for the different lower bounds (expressed as average degree). Note also the fact that the lower and upper quartile separations are fairly narrow and the outliers are few.

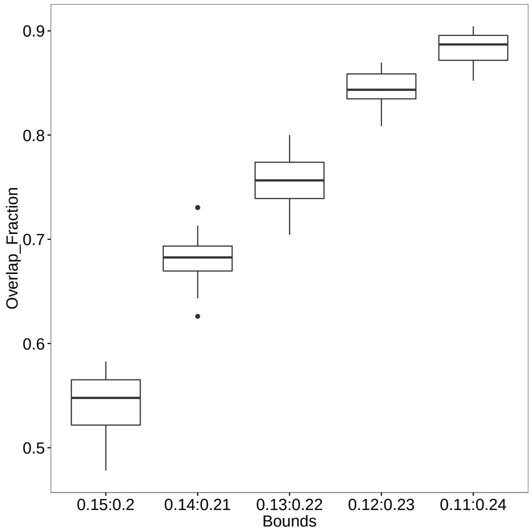

Figure 17: Boxplot of the distribution of overlap in directed edges between the network G on the full dataset for the absolute interval (0.15,0.2) of CIF measure and that constructed from the subsampled dataset for each of the intervals shown on the x-axis. As in the previous case, the interquartile range is about 0.05, and there are very few outliers.

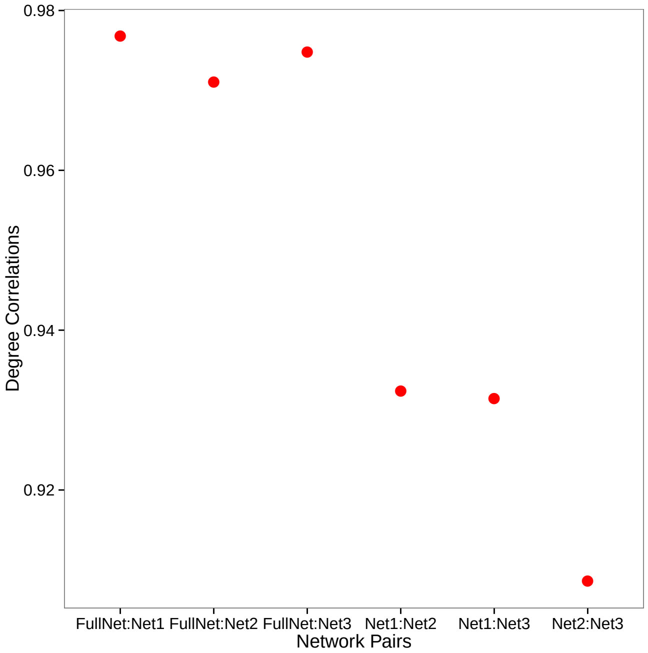

Figure 18: Vertex degree correlation between different pairs of networks. FullNet refers to the network on the full interval (1,4).

K.1 Stability with respect to the thresholds

We next consider stability around the choice of thresholds for the collapsed network shown in Fig. 3 of the main manuscript. We first note the obvious fact that two disjoint intervals of thresholds will, by definition, lead to networks with no common edge. Thus network similarity metrics based on edge comparisons such as the Jaccard index would be unsuitable for our purposes. We seek independent methods to establish the reliability of the general structure and communities that have been shown. Consider an interval range in the thresholds (once again, specified using average degree) given by (GLB,GUB) and subdivide that interval into n equal subintervals of width $\Delta = \frac{GLB-GUB}{n}$. We construct distinct networks with each of these $n+1$ intervals (including the full interval). In each of these networks, as earlier, there is no isolated vertex. The following two methods are then applied to these networks:

(a) We determine the nodes that are common to all the $n+1$ networks. We then consider the correlation in vertex degrees between every pair of networks. We examine the case, GLB=4,GUB=1, and $n = 3$ (we set the parameters with the aim that the final network shown in the manuscript between thresholds 1 and 2 lies in that interval). Fig. 18 shows a plot of the correlation for the different pairs - Net 1, 2 and 3 correspond to intervals (1,2), (2,3) and (3,4). We find that the correlations are all over 0.9.

(b) To understand how robust the community structure is, we use the Walktrap community detection algorithm [7] on the network with the full range (once again

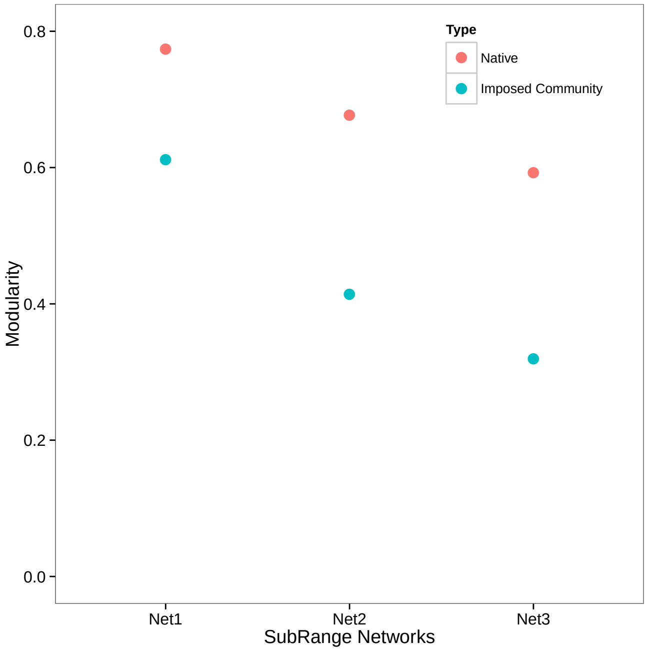

Figure 19: Imposed refers to modularity scores for the community assignment based on the community detection algorithm applied to the network on the full range (1,4). Native refers to modularity scores obtained by applying the algorithm directly to the network. As before, Net 1,2 and 3 refers to sub-intervals (1,2), (2,3) and (3,4) respectively.

GLB=4,GUB=1) and obtain a grouping of vertices into distinct communities. We then impose this community structure on each of the three networks on the sub-intervals and compute the corresponding modularity score and compare that to the modularity score obtained by independently applying the Walktrap algorithm to each. This is shown in Fig. 19. We find that the imposed modularity scores are all over 0.3, and more than 50% of what is obtained when independent community detection is applied.

We also note that the heat map in Fig. 11, also a vertex-degree correlation, also offers evidence for the stability of the networks across different thresholds (vertices in the figure are full 3-character ICD 10 coded diseases).

L Disease Prevalence Distribution

The distribution of the prevalences of the different diseases in the full cohort and in the subset we considered is shown in Fig. 20.

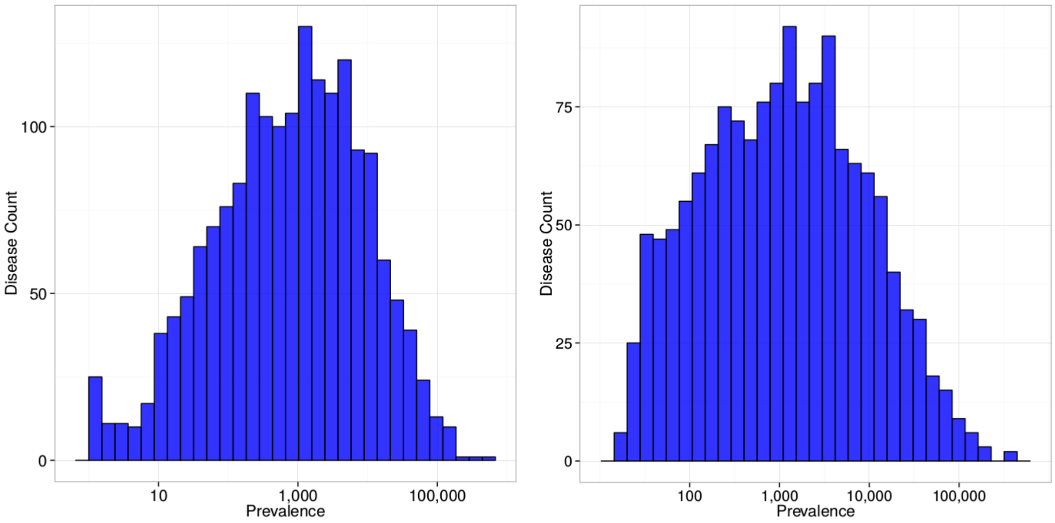

Figure 20: Distribution of the prevalences among (a) all ICD 10 three character diseases in the cohort (b) the subset under consideration in our analysis, which excludes codes starting with either V,W,X,Y or Z, and prevalences less than 20.

M Disease Cluster Codes

ICD 10 Chapter Title of Diseases Clusters: A0-B9: Infectious and Parasitic diseases; **C0-D4**: Neoplasms; **D5-D9**: Diseases of the blood-forming organs and immune mechanism disorders; **E0-E9**: Endocrine, nutritional and metabolic diseases; **F0-F9**: Mental and behavioral disorders; **G0-G9**: Diseases of the nervous system; **H0-H5**: Diseases of the eye and adnexa; **H6-H9**: Diseases of the ear and mastoid process; **I0-I9**: Diseases of the circulatory system; **J0-J9**: Diseases of the respiratory system; **K0-K9**: Diseases of the digestive system; **L0-L9**: Diseases of the skin and subcutaneous tissue; **M0-M9**: Diseases of the musculoskeletal system and connective tissue; **N0-N9**: Diseases of the genitourinary system; **O0-O9**: Pregnancy, childbirth and the puerperium; **P0-P9**: Certain conditions originating in the perinatal period; **Q0-Q9**: Congenital malformations, deformations and chromosomal abnormalities; **R0-R9**: Symptoms, signs and abnormal clinical and laboratory findings, not elsewhere classified; **S0-T9**: Injury, poisoning and certain other consequences of external causes.

ICD 10 code of Diseases clusters shown in Fig3	
Number	Cluster members ICD 10 Code
1	C0, E3, P8, R1, C1
2	N1, I6, G8, R7, Q0, Q2, G9, G4, I7, N0, L9, E1, T8
3	O6, O4, O3, O2, O1, P9, O9, P0, O0, O7
4	B1, K7, C8, D7, K9, D6, R5, K2
5	R9, D3, C7, C3
6	D2, N9, P6, N8
7	T5, T3, F3, F9, F5, F2, F4, F1
8	A4, L8, B4, P3
9	M3, R4, I4, I3, M1, I2, E2, I5, I9, I1, R0, E7, I0, R8, H8
10	J4, J8, B5, A3, B2, J6, G7, J9, G1, J2, J7, Q9, J1
11	A5, O8
12	U8, B9
13	K5, K6
14	G3, R3, G2, N3
15	T0, S1, S0
16	H4, H3
17	F6, T4

Table 3: Listing of diseases in each cluster shown in Fig. 3 of the main text

Table 3 (diseases with significantly higher incidence in men)

ICD10	Normalized gender difference	Disease Name
'N35'	0,88291	'Urethral stricture'
'J92'	0,83184	'Pleural plaque'
'K40'	0,82368	'Inguinal hernia'
'N32'	0,76143	'Other disorders of bladder'
'N21'	0,7513	'Calculus of lower urinary tract'
'S97'	0,7202	'Crushing injury of ankle and foot'
'R39'	0,70488	'Oth and unsp symptoms and signs involving the GU sys'
'T26'	0,6992	'Burn and corrosion confined to eye and adnexa'
'S98'	0,67516	'Traumatic amputation of ankle and foot'
'S68'	0,67415	'Traumatic amputation of wrist, hand and fingers'
'S21'	0,67163	'Open wound of thorax'
'F18'	0,65536	'Inhalant related disorders'
'S56'	0,64238	'Injury of muscle, fascia and tendon at forearm level'
'S05'	0,6364	'Injury of eye and orbit'
'F12'	0,63234	'Cannabis related disorders'
'T35'	0,62878	Frostbite involving multiple body regions and unspecified frostbite
'T34'	0,62532	'Frostbite with tissue necrosis'
'S76'	0,62007	'Injury of muscle, fascia and tendon at hip and thigh level'
'C32'	0,61929	'Malignant neoplasm of larynx'
'S67'	0,60925	'Crushing injury of wrist, hand and fingers'
'S86'	0,59794	'Injury of muscle, fascia and tendon at lower leg level'
'S46'	0,59607	'Injury of muscle, fascia and tendon at shldr/up arm'
'S57'	0,58859	'Crushing injury of elbow and forearm'
'T70'	0,58698	'Effects of air pressure and water pressure'
'S65'	0,58679	'Injury of blood vessels at wrist and hand level'
'B22'	0,58667	Human immunodeficiency virus [HIV] disease resulting in other specified diseases
'S55'	0,58654	'Injury of blood vessels at forearm level'
'S66'	0,58572	'Injury of muscle, fascia and tendon at wrist and hand level'

'C13'	0,58337	'Malignant neoplasm of hypopharynx'
'M72'	0,57547	Fibroblastic disorders'
'D02'	0,5685	'Carcinoma in situ of middle ear and respiratory system'
'R31'	0,55967	'Hematuria'
'B20'	0,55878	'Human immunodeficiency virus [HIV] disease'
'T23'	0,55693	'Burn and corrosion of wrist and hand'
'S69'	0,54903	'Other and unspecified injuries of wrist, hand and finger(s)'
'C12'	0,54867	Malignant neoplasm of pyriform sinus'
'C45'	0,54763	Mesothelioma'
'T20'	0,54609	'Burn and corrosion of head, face, and neck'
'C15'	0,54573	Malignant neoplasm of esophagus'
'S41'	0,53811	'Open wound of shoulder and upper arm'
'S11'	0,53542	'Open wound of neck'
'S61'	0,53018	'Open wound of wrist, hand and fingers'
'I71'	0,52732	'Aortic aneurysm and dissection'
'C67'	0,52559	'Malignant neoplasm of bladder'
'T58'	0,52345	'Toxic effect of carbon monoxide'
'B24'	0,52286	Unspecified human immunodeficiency virus [HIV] disease
'M10'	0,52256	'Gout'
'T31'	0,52011	'Burns classified accord extent body involv'
'T52'	0,51294	'Toxic effect of organic solvents'
'S71'	0,50853	'Open wound of hip and thigh'
'T25'	0,5072	'Burn and corrosion of ankle and foot'
'L05'	0,5006	'Pilonidal cyst and sinus'
'I30'	0,50033	Acute pericarditis'
'D09'	0,49867	'Carcinoma in situ of other and unspecified sites'
'S38'	0,4967	'Crush inj & traum amp of abd,low back, pelv & extrn genitals'
'T22'	0,49517	'Burn and corrosion of shldr/up lmb, except wrist and hand'
'I40'	0,4951	'Acute myocarditis'
'T68'	0,49502	'Hypothermia'
'S64'	0,48949	'Injury of nerves at wrist and hand level'
'S91'	0,48641	'Open wound of ankle, foot and toes'
'S77'	0,48581	'Crushing injury of hip and thigh'
'S14'	0,48436	'Injury of nerves and spinal cord at neck level'

'S45'	0,48403	'Injury of blood vessels at shoulder and upper arm level'
'G47'	0,48147	Sleep disorders'
'B23'	0,48135	Human immunodeficiency virus [HIV] disease resulting in other conditions
'S24'	0,48043	'Injury of nerves and spinal cord at thorax level'
'I72'	0,47483	'Other aneurysm'
'T29'	0,47439	Burns of multiple regions, unspecified degree
'S85'	0,46947	'Injury of blood vessels at lower leg level'
'S62'	0,46694	'Fracture at wrist and hand level'
'S88'	0,46557	'Traumatic amputation of lower leg'
'J34'	0,46438	'Other and unspecified disorders of nose and nasal sinuses'
'T08'	0,46359	Fracture of spine, level unspecified
'T75'	0,4537	'Other and unspecified effects of other external causes'
'S54'	0,44918	'Injury of nerves at forearm level'
'I86'	0,44583	'Varicose veins of other sites'
'I42'	0,44359	'Cardiomyopathy'
'F15'	0,44294	'Other stimulant related disorders'
'T30'	0,44268	'Burn and corrosion, body region unspecified'
'S02'	0,44121	'Fracture of skull and facial bones'
'S27'	0,43982	'Injury of other and unspecified intrathoracic organs'
'C09'	0,43953	'Malignant neoplasm of tonsil'
'F14'	0,43937	'Cocaine related disorders'
'T27'	0,43906	'Burn and corrosion of respiratory tract'
'S96'	0,43896	'Injury of muscle and tendon at ankle and foot level'
'F16'	0,43749	'Hallucinogen related disorders'
'I22'	0,43486	'Subsequent STEMI & NSTEMI mocard infrc'
'S12'	0,43182	'Fracture of cervical vertebra and other parts of neck'
'F10'	0,43164	'Alcohol related disorders'
'I25'	0,43016	'Chronic ischemic heart disease'
'I98'	0,42956	Other disorders of circulatory system in diseases classified elsewhere
'C14'	0,42612	'Malig neoplasm of sites in the lip, oral cavity and pharynx'
'I23'	0,42522	'Certain crnt comp fol STEMI & NSTEMI mocard infrc <= 28 day'
'K70'	0,42316	'Alcoholic liver disease'

'G32'	0,42138	'Oth degeneratv disord of nervous sys in dis classd elswhr'
'D66'	0,42084	'Hereditary factor VIII deficiency'
'S51'	0,4198	'Open wound of elbow and forearm'
'F95'	0,41853	'Tic disorder'
'S87'	0,41816	'Crushing injury of lower leg'
'I85'	0,41642	'Esophageal varices'
'S49'	0,41636	'Other and unspecified injuries of shoulder and upper arm'
'C10'	0,41275	Malignant neoplasm of oropharynx'
'I21'	0,41261	'STEMI & NSTEMI mocard infrc'
'A52'	0,4124	'Late syphilis'
'S31'	0,40929	'Opn wnd abdomen, lower back, pelvis and external genitals'
'S75'	0,40557	'Injury of blood vessels at hip and thigh level'
'A79'	0,40538	'Other rickettsioses'
'J68'	0,4035	'Resp cond d/t inhalation of chemicals, gas, fumes and vapors'
'S99'	0,40176	'Other and unspecified injuries of ankle and foot'
'S07'	0,40044	'Crushing injury of head'

Table 4 (diseases with significantly higher incidence in women)

'C50'	0,98916	'Malignant neoplasm of breast'
'D24'	0,97613	'Benign neoplasm of breast'
'N61'	0,957653	'Inflammatory disorders of breast'
'N64'	0,944543	'Other disorders of breast'
'N62'	0,894588	'Hypertrophy of breast'
'F50'	0,841771	'Eating disorders'
'E65'	0,831109	'Localized adiposity'
'M32'	0,729727	'Systemic lupus erythematosus (SLE)'
'A60'	0,726526	'Anogenital herpesviral [herpes simplex] infections'
'D07'	0,714052	'Carcinoma in situ of other and unspecified genital organs'
'M81'	0,695418	'Osteoporosis without current pathological fracture'
'E04'	0,684815	'Other nontoxic goiter'
'E03'	0,680651	'Other hypothyroidism'
'Q79'	0,670293	'Congenital malformations of musculoskeletal system, NEC'
'L93'	0,661398	'Lupus erythematosus'
'L91'	0,650627	'Hypertrophic disorders of skin'
'Q65'	0,648336	'Congenital deformities of hip'
'E06'	0,637087	'Thyroiditis'
'R15'	0,636846	'Fecal incontinence'
'H06'	0,630302	Disorders of lacrimal system and orbit in diseases classified elsewhere
'M18'	0,625935	'Osteoarthritis of first carpometacarpal joint'
'E05'	0,624047	'Thyrotoxicosis [hyperthyroidism]'
'A63'	0,619512	'Oth predominantly sexually transmitted diseases, NEC'
'M20'	0,607645	'Acquired deformities of fingers and toes'
'D20'	0,601721	'Benign neoplm of soft tissue of retroperiton and peritoneum'
'M80'	0,600104	'Osteoporosis with current pathological fracture'
'M15'	0,598912	'Polyosteoarthritis'
'D34'	0,596154	'Benign neoplasm of thyroid gland'
'M34'	0,587831	'Systemic sclerosis [scleroderma]'

'D22'	0,587496	'Melanocytic nevi'
'L90'	0,58442	'Atrophic disorders of skin'
'E07'	0,583707	'Other disorders of thyroid'
'K66'	0,55775	'Other disorders of peritoneum'
'K58'	0,538944	'Irritable bowel syndrome'
'G43'	0,530097	'Migraine'
'D19'	0,511061	'Benign neoplasm of mesothelial tissue'
'D56'	0,503142	'Thalassemia'
'E02'	0,501008	'Subclinical iodine-deficiency hypothyroidism'
'E21'	0,49124	'Hyperparathyroidism and other disorders of parathyroid gland'
'M08'	0,481207	'Juvenile arthritis'
'E24'	0,475066	'Cushings syndrome'
'M05'	0,473671	'Rheumatoid arthritis with rheumatoid factor'
'E73'	0,470756	'Lactose intolerance'
'D32'	0,4673	'Benign neoplasm of meninges'
'Q44'	0,467142	'Congenital malform of gallbladder, bile ducts and liver'
'E68'	0,466254	'Sequelae of hyperalimentation'
'C23'	0,462738	'Malignant neoplasm of gallbladder'
'E01'	0,462147	'Iodine-deficiency related thyroid disorders and allied cond'
'M06'	0,459163	'Other rheumatoid arthritis'
'E89'	0,45848	'Postproc endocrine and metabolic comp and disorders, NEC'
'L94'	0,456585	'Other localized connective tissue disorders'
'M09'	0,447544	Juvenile arthritis in diseases classified elsewhere
'N99'	0,441951	'Intraop and postproc comp and disorders of GU sys, NEC'
'M41'	0,434185	'Scoliosis'
'L63'	0,434012	Alopecia areata'
'M94'	0,433137	'Other disorders of cartilage'
'M35'	0,432171	'Other systemic involvement of connective tissue'
'R32'	0,423567	'Unspecified urinary incontinence'
'G97'	0,411692	'Intraop and postproc comp and disorders of nervous sys, NEC'
'L43'	0,40214	'Lichen planus'

References

[1] A. Hidalgo and N. Blumm, "A Dynamic Network Approach for the Study of Human Phenotypes," *PLoS Comput. Biol.*, vol. 5, p. e1000353, apr 2009.

[2] J. Zhang and F. Y. Kai, "What's the relative risk?," *JAMA J. Am. Med. Assoc.*, vol. 280, pp. 1690–1691, nov 1998.

[3] S. A. Ahmed, B. D. Hissong, D. Verthelyi, K. Donner, K. Becker, and E. Karpuzoglu-Sahin, "Gender and risk of autoimmune diseases: Possible role of estrogenic compounds," *Environ. Health Perspect.*, vol. 107, pp. 681–686, oct 1999.

[4] J. Olivier and M. L. Bell, "Effect Sizes for 2??2 Contingency Tables," *PLoS One*, vol. 8, p. e58777, jan 2013.

[5] A. B. Jensen, P. L. Moseley, T. I. Oprea, S. G. Ellesøe, R. Eriksson, H. Schmock, P. B. Jensen, L. J. Jensen, and S. Brunak, "Temporal disease trajectories condensed from population-wide registry data covering 6.2 million patients.," *Nat. Commun.*, vol. 5, p. 4022, jan 2014.

[6] A. L. Franks and J. E. Slansky, "Multiple associations between a broad spectrum of autoimmune diseases, chronic inflammatory diseases and cancer," *Anticancer Res.*, vol. 32, pp. 1119–1136, apr 2012.

[7] P. Pons and M. Latapy, "Computing Communities in Large Networks Using Random Walks," *Phys. Rev. E*, vol. 10, no. 2, pp. 284–293, 2005.
